# Supplementary figures and images for: Abnormal accumulation of lipid droplets in neurons induces the conversion of alpha-Synuclein to proteolytic resistant forms in a Drosophila model of Parkinson’s disease
Source: PLoS Genet. 2021 Nov 17;17(11):e1009921. doi: 10.1371/journal.pgen.1009921 (PMC8635402; doi:10.1371/journal.pgen.1009921)

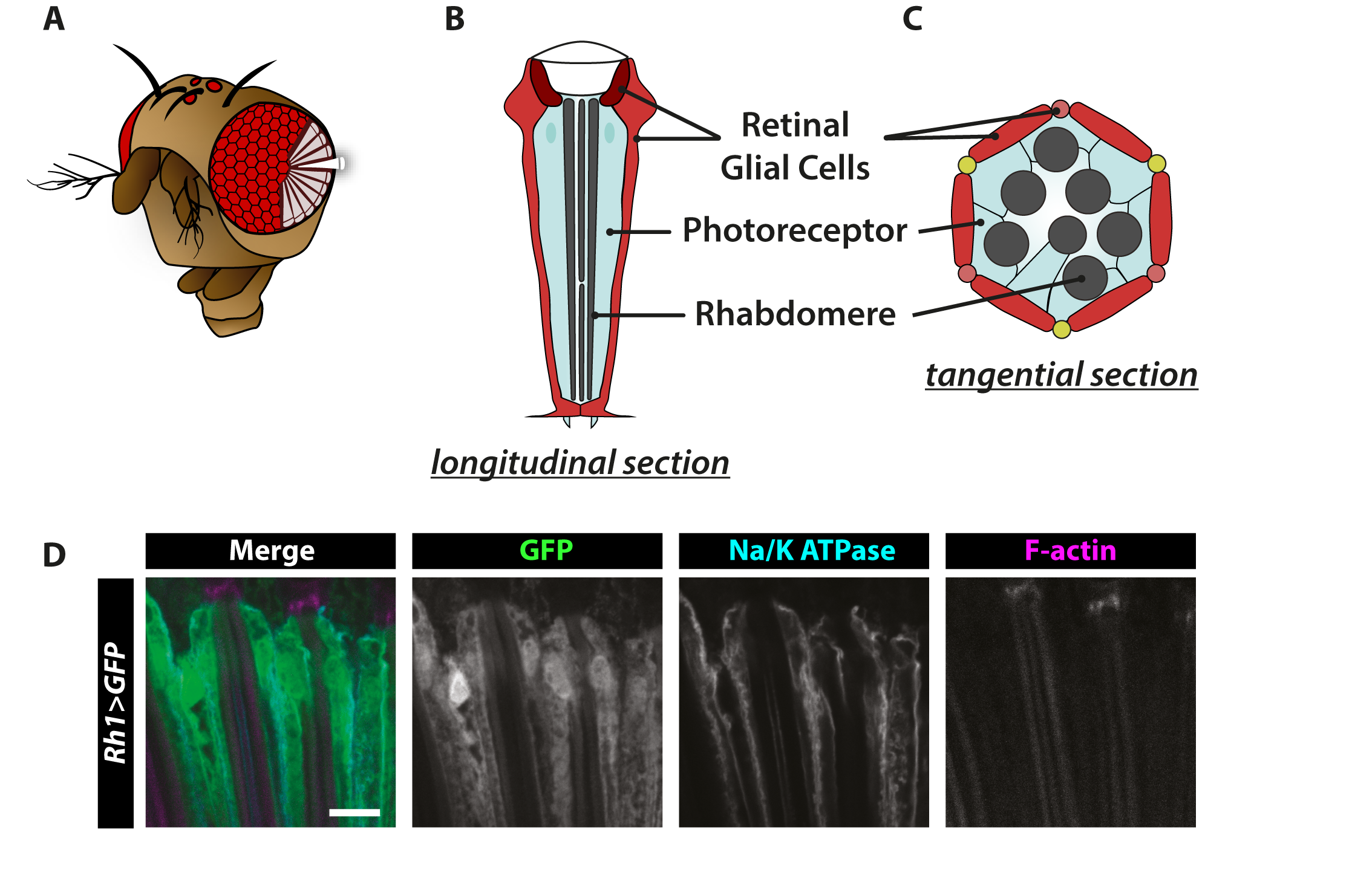

Supplement: S1 Fig — (A) Diagram of a Drosophila compound eye. The eye is composed of about 800 repeated units called ommatidia (hexagonal shapes). Longitudinal sections (shown in white) of the ommatidia allow visualization of the retinal cells that span the entire width of the retina. (B) Diagram of a longitudinal section of one ommatidium. Each ommatidium is composed of 8 photoreceptor neurons (light blue), each containing one rhabdomere (dark gray), and glial cells (also known as primary, secondary and tertiary retinal pigment cells; maroon, medium red and, light red respectively) that are juxtaposed to photoreceptor neurons from the apical to the basal retina. (C) Diagram of a cross-section of one ommatidium. In addition to the glial cells (2 primary, 6 secondary [medium red] and 3 tertiary [light red] pigment cells), each ommatidium contains 3 bristle cells (yellow) originating from the neuronal lineage. (D) Immunostaining of whole-mount retinas from flies expressing GFP in photoreceptor (Rh1-GAL4). Photoreceptor plasma membranes are in cyan (anti-Na+/K+ ATPase) and rhabdomeres are in magenta (phalloidin-rhodamine). GFP is visible only in the photoreceptor cytoplasm. Scale bar, 10μm. (TIF) [file pgen.1009921.s001.tif]

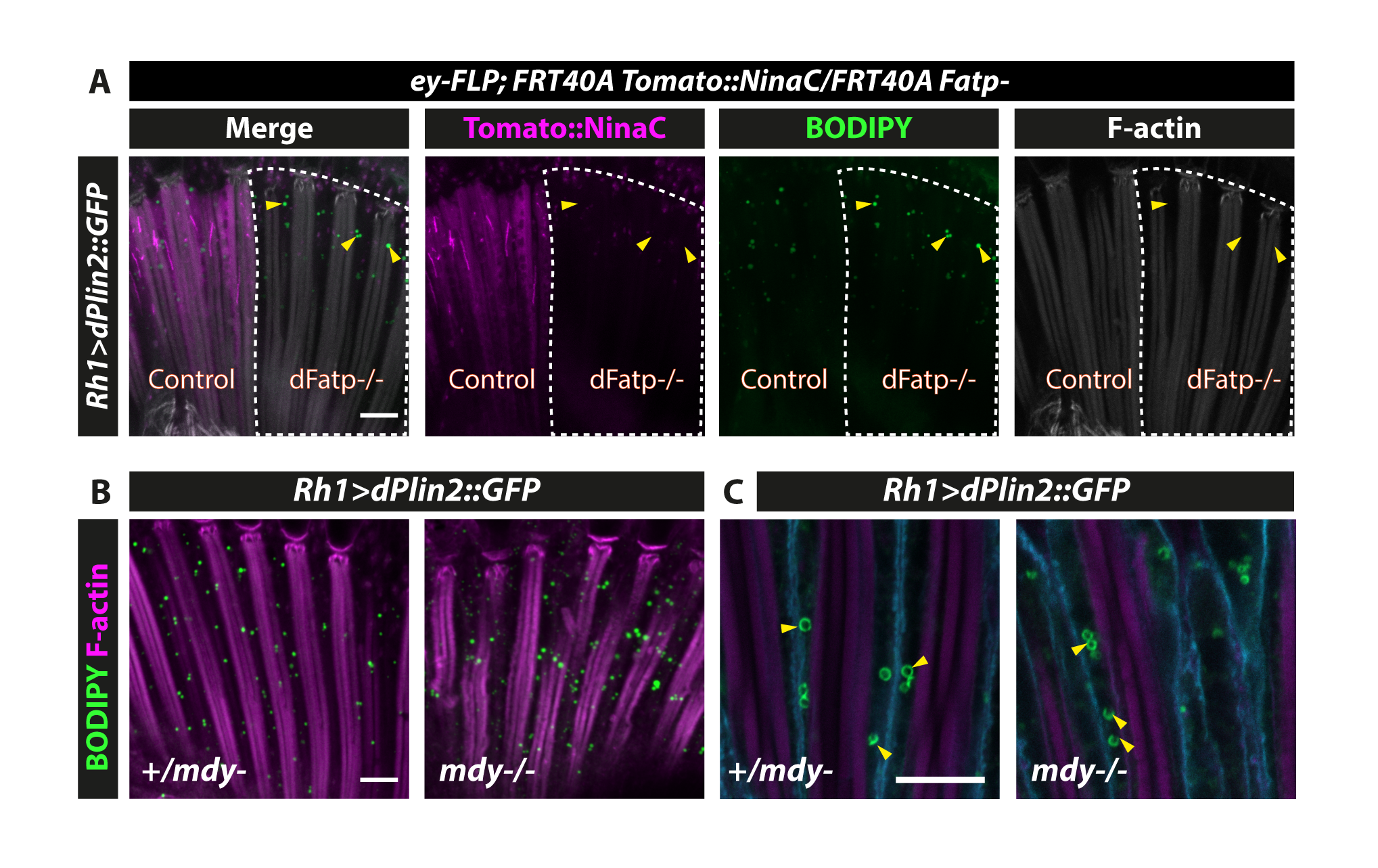

Supplement: S2 Fig — (A) LD staining of whole-mount retinas from flipase mediated FRT40A dFatpk10307 mutant clone in conjunction with expression of dPlin2::GFP in photoreceptors (Rh1-GAL4). LDs are in green (BODIPY), wild type photoreceptors are in magenta (FRT40A tdTomato::NinaC) and rhabdomeres are in grey (phalloidin-rhodamine labeling of F-actin). LDs are visible in photoreceptors lacking dFatp (mutant clone surrounded by dashed line). Scale bar, 10 μm. (B, C) LD staining of whole-mount retinas from mdyQX25 heterozygous and homozygous flies expressing dPlin2::GFP in photoreceptors (Rh1-GAL4). Scale bar, 10 μm.(B) LDs are in green (BODIPY), and rhabdomeres are in magenta (phalloidin-rhodamine). (C) Photoreceptor plasma membranes are in cyan (anti-Na+/K+ ATPase) and rhabdomeres are in magenta (phalloidin-rhodamine) dPlin2::GFP stains LDs as a ring shape in the photoreceptor cytoplasm (yellow arrowheads). (TIF) [file pgen.1009921.s002.tif]

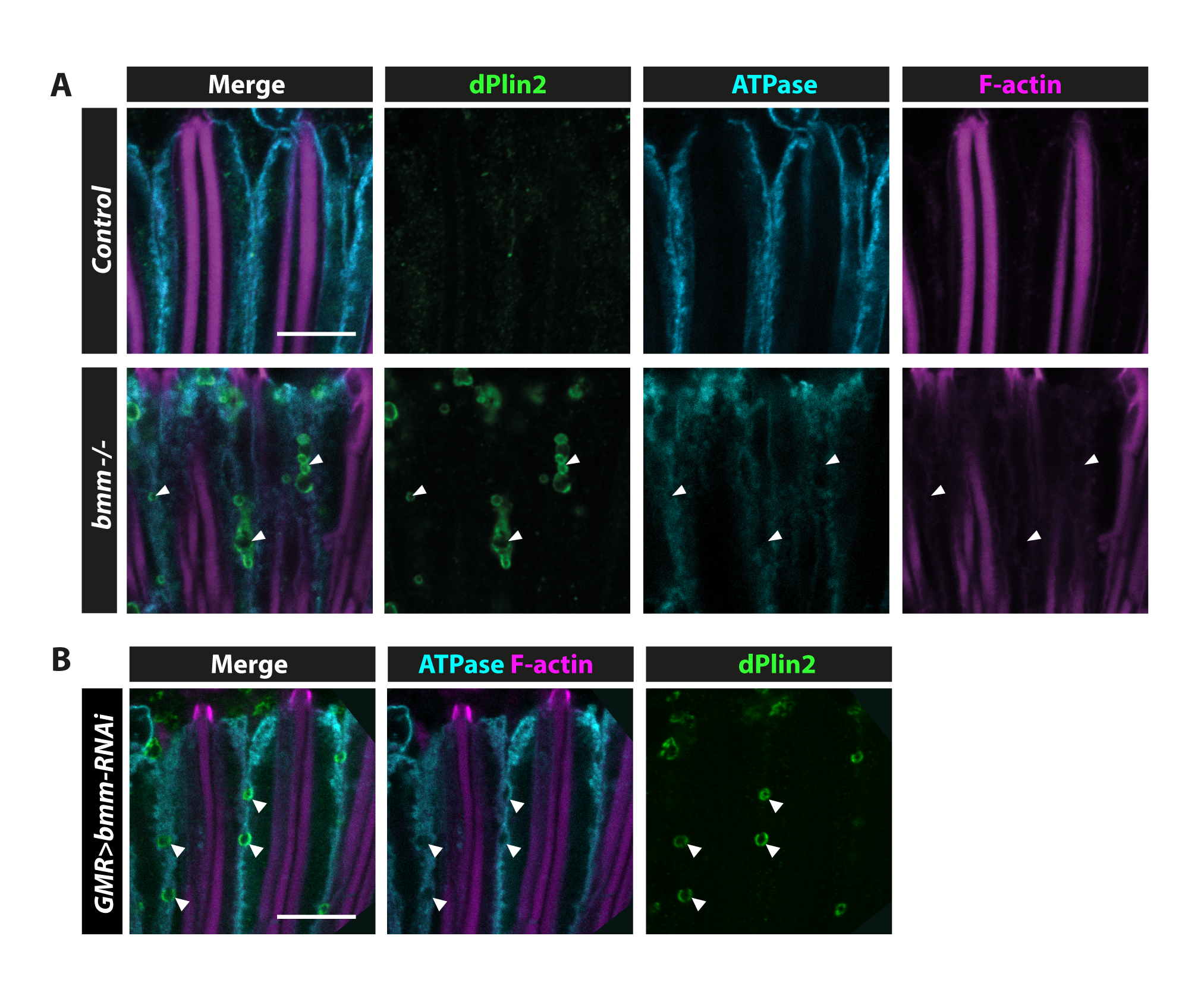

Supplement: S3 Fig — (A) Immunostaining of whole-mount retinas from control (w1118) or homozygous bmm1 mutant flies. LDs are in green (anti-dPlin2), photoreceptor plasma membranes are in cyan (anti-Na+/K+ ATPase) and rhabdomeres are in magenta (phalloidin-rhodamine). Scale bar, 10 μm. (B) Immunostaining of whole-mount retinas from flies expressing RNAi targeting bmm lipase under the control of the pan-retinal driver GMR-GAL4. LDs are visible in green (anti-dPlin2), photoreceptor plasma membranes are in cyan (anti-Na+/K+ ATPase) and rhabdomeres are in magenta (phalloidin-rhodamine). Scale bar, 10 μm. (TIF) [file pgen.1009921.s003.tif]

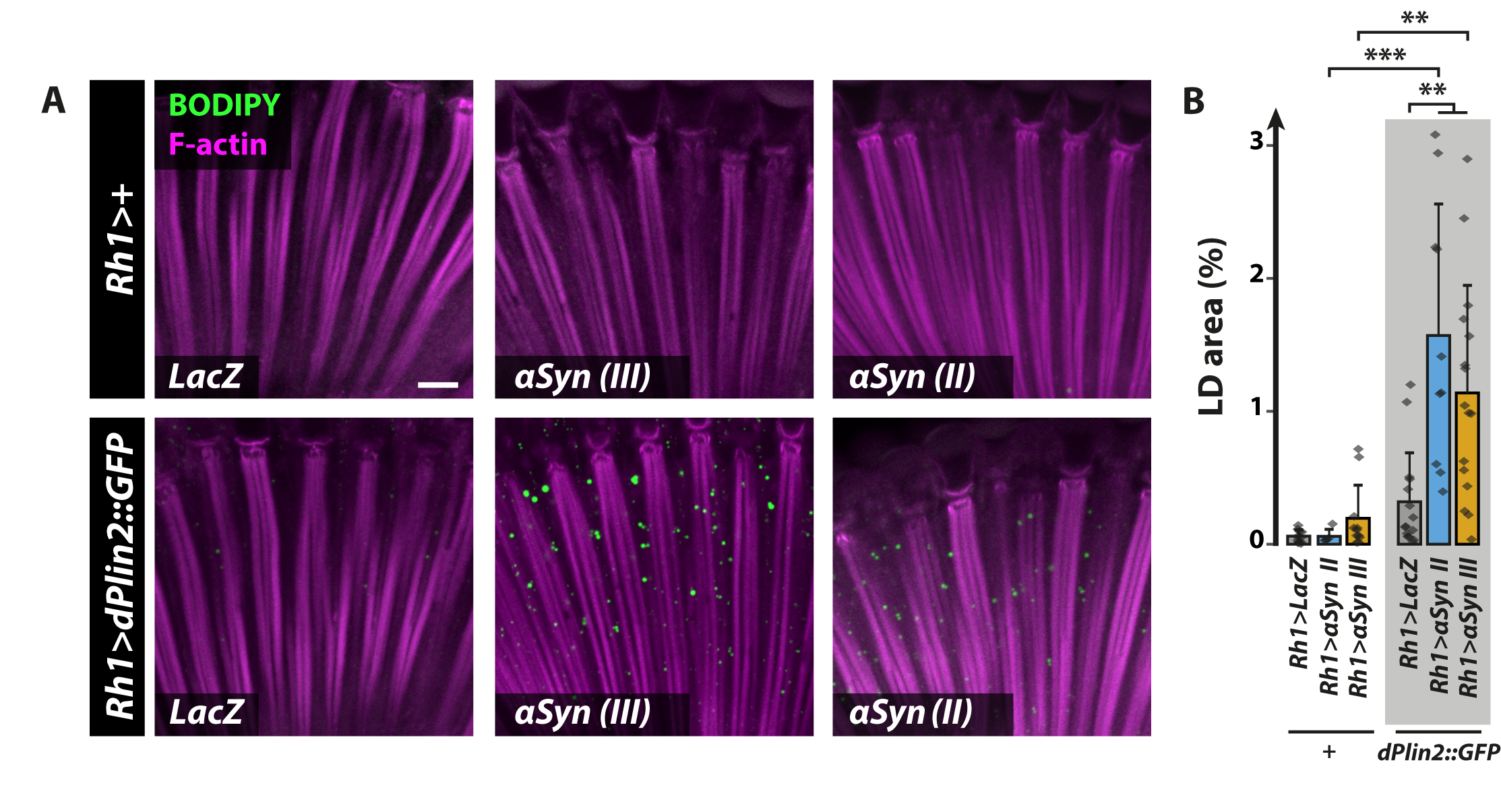

Supplement: S4 Fig — (A) LD staining of whole-mount retinas from flies with photoreceptor neuron-specific expression of LacZ (control) or αSynWT (two independent lines located on second [II] and third chromosome [III]; the line αSyn III is used elsewhere in the article) alone or in conjunction with dPlin2::GFP. LDs are shown in green (BODIPY) and photoreceptor rhabdomeres are shown in magenta (phalloidin-rhodamine). Scale bar, 10 μm. (B) Quantification of LD area from the images shown in (A). Mean ± SD. ***p<0.001, **p<0.01 by ANOVA with Tukey’s HSD test. (TIF) [file pgen.1009921.s004.tif]

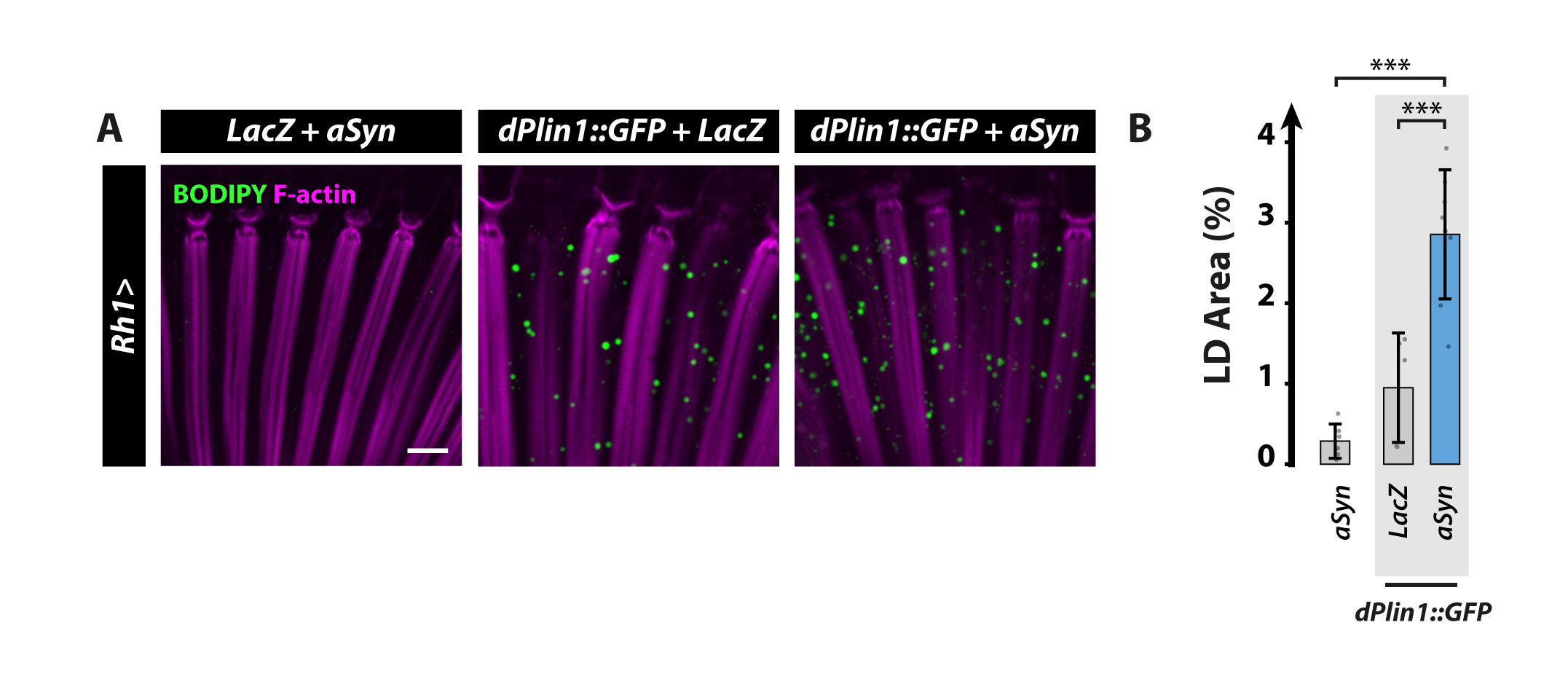

Supplement: S5 Fig — (A) LD staining of whole-mount retinas from flies expressing LacZ (control) or human αSyn alone or in conjunction with dPlin1::GFP in photoreceptor neurons (Rh1-GAL4). LDs are in green (BODIPY) and photoreceptor rhabdomeres are in magenta (phalloidin-rhodamine). Scale bar, 10 μm. (B) Quantification of LD area from images shown in (A). Mean ± SD. ***p<0.001 by ANOVA with Tukey’s HSD test. (TIF) [file pgen.1009921.s005.tif]

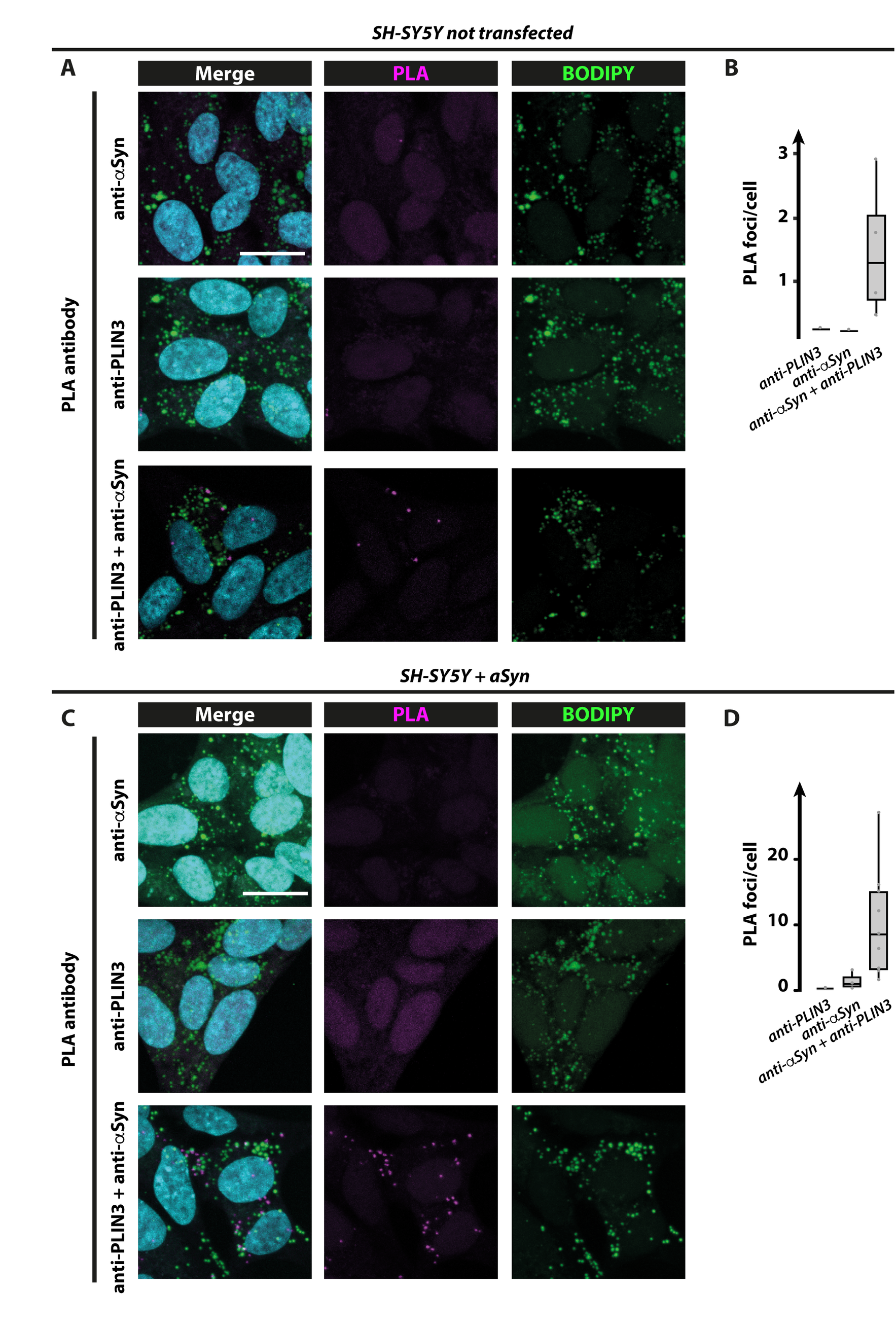

Supplement: S6 Fig — (A). Proximity ligation assay between αSyn and PLIN3 in non-transfected SH-SY5Y cells. The PLA signal generated by close proximity of the two protein-bound primary antibodies shown in magenta is not visible when antibody against PLIN3 or αSyn are used separately. LDs are in green (BODIPY), and nuclei are counterstained with DAPI (cyan). Scale bars, 15 μm. (B). Quantification of the number of PLA foci per cell seen in (A). (C). Proximity ligation assay between αSyn and PLIN3 in transfected SH-SY5Y cells with αSynWT. The PLA signal generated by close proximity of the two protein-bound primary antibodies shown in magenta is not visible when antibody against PLIN3 or αSyn are used separately. LDs are in green (BODIPY), and nuclei are counterstained with DAPI (cyan). Scale bars, 15 μm. (D). Quantification of the number of PLA foci per cell seen in (C). (TIF) [file pgen.1009921.s006.tif]

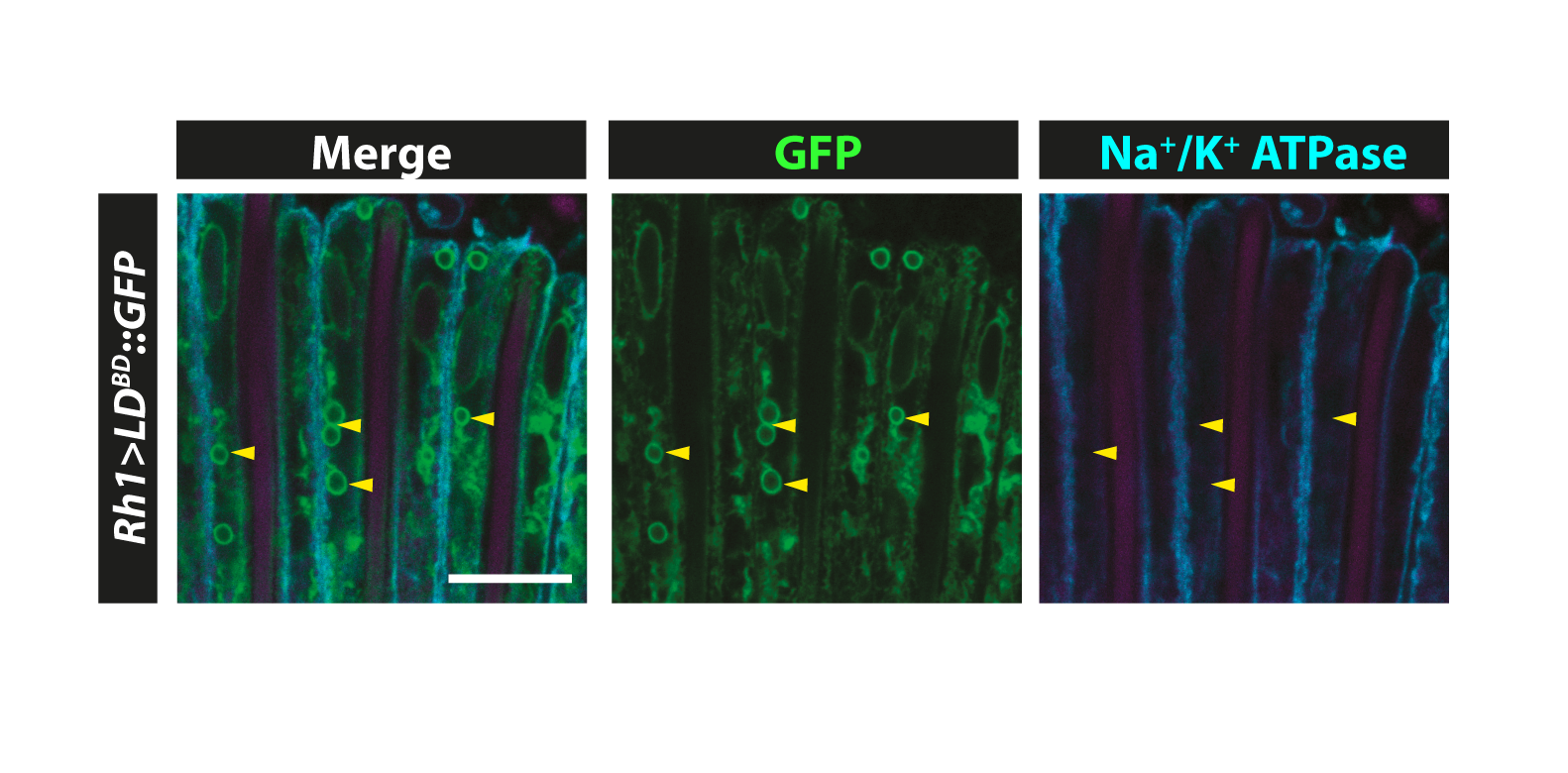

Supplement: S7 Fig — Immunostaining of whole-mount retinas from flies with photoreceptor neuron-specific expression of the minimal LD-binding domain of Klarsicht fused to GFP (UAS-LDBD::GFP) [57]. GFP localizes to the ring-shaped structures (yellow arrowheads) located between photoreceptor plasma membranes in cyan (anti-Na+/K+ ATPase) and rhabdomere in magenta (phalloidin-rhodamine). Scale bar, 10 μm. (TIF) [file pgen.1009921.s007.tif]

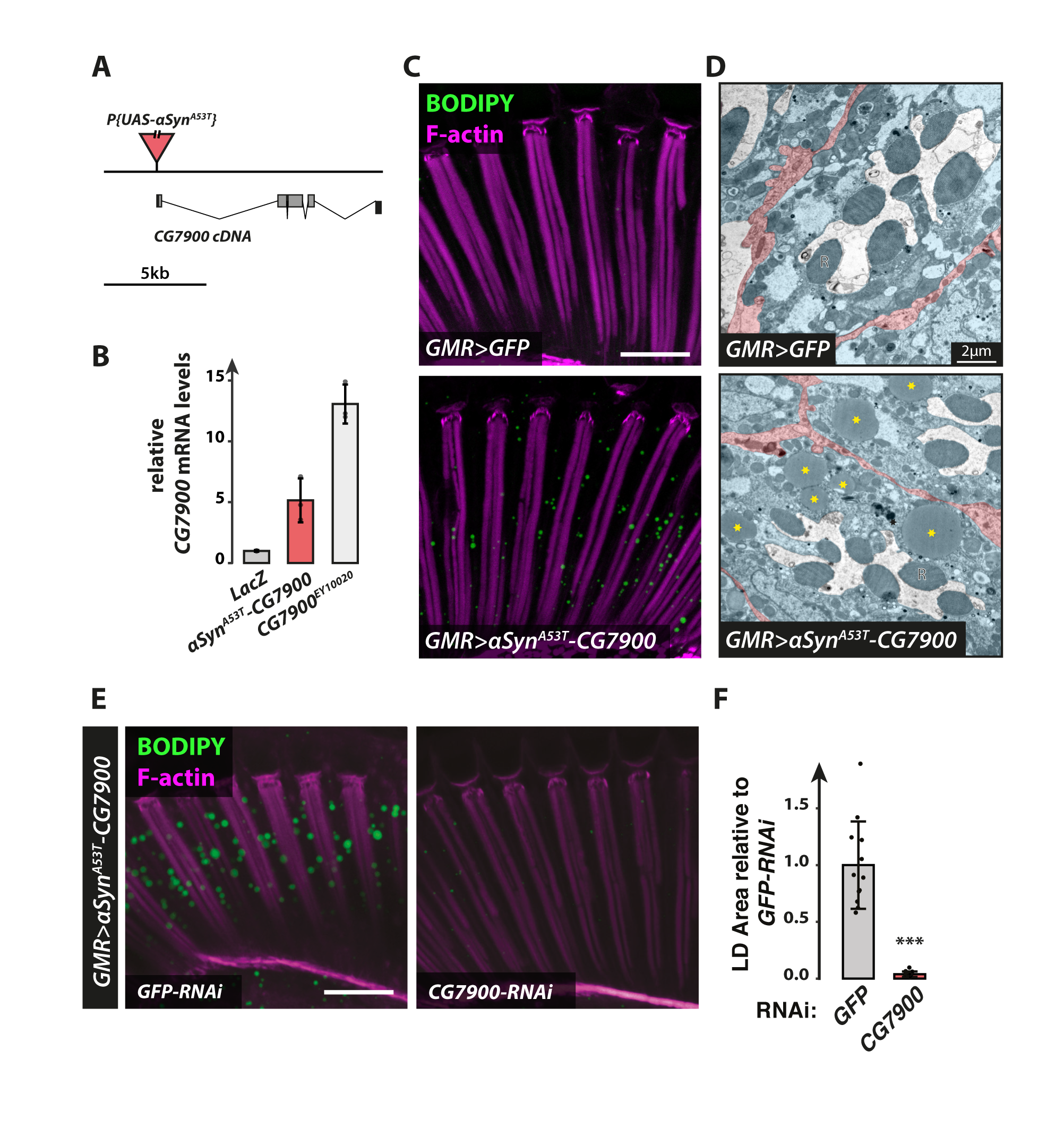

Supplement: S8 Fig — (A) Diagram of the genomic localization of the P{UAS-αSynA53T} transgene mapped using the Splinkerette protocol. The P-element carrying the upstream activating sequence (UAS) upstream of the coding sequence of human αSynA53T is inserted in the promoter region of CG7900. (B) RT-qPCR analysis of CG7900 mRNA in heads of flies expressing LacZ, αSynA53T-CG7900, or CG7900 (EP-[UAS] insertion, EY10020) under the control of the pan-retinal driver GMR-GAL4. mRNA levels are expressed as the mean ± SD of triplicates relative to the level in control (GMR>LacZ) flies. (C) LD staining of whole-mount retinas from flies with pan-retinal expression of GFP or αSynA53T-CG7900. LDs are shown in green (BODIPY) and photoreceptor rhabdomeres are in magenta (phalloidin-rhodamine). Scale bar, 20 μm. (D) TEM images of ommatidia cross-sections from 60-day-old flies with pan-retinal expression of GFP (top panel) or αSynA53T-CG7900 (bottom panel). Each panel shows a representative cross-section of one ommatidium containing seven photoreceptors (false-colored blue) with central rhabdomeres (R) surrounded by retinal glial cells (false-colored orange). Yellow asterisks indicate LDs accumulating in the photoreceptor cytoplasm of flies expressing αSynA53T-CG7900. Scale bar, 2 μm. (E) LD staining of whole-mount retinas from flies expressing αSynA53T-CG7900 in conjunction with GFP-RNAi or CG7900-RNAi in photoreceptors (GMR-GAL4). LDs are shown in green (BODIPY) and photoreceptor rhabdomeres are in magenta (phalloidin-rhodamine). Scale bar, 20 μm. (F) Quantification of LD area from the images shown in (E). Mean ± SD. ***p<0.001 by t-test. (TIF) [file pgen.1009921.s008.tif]

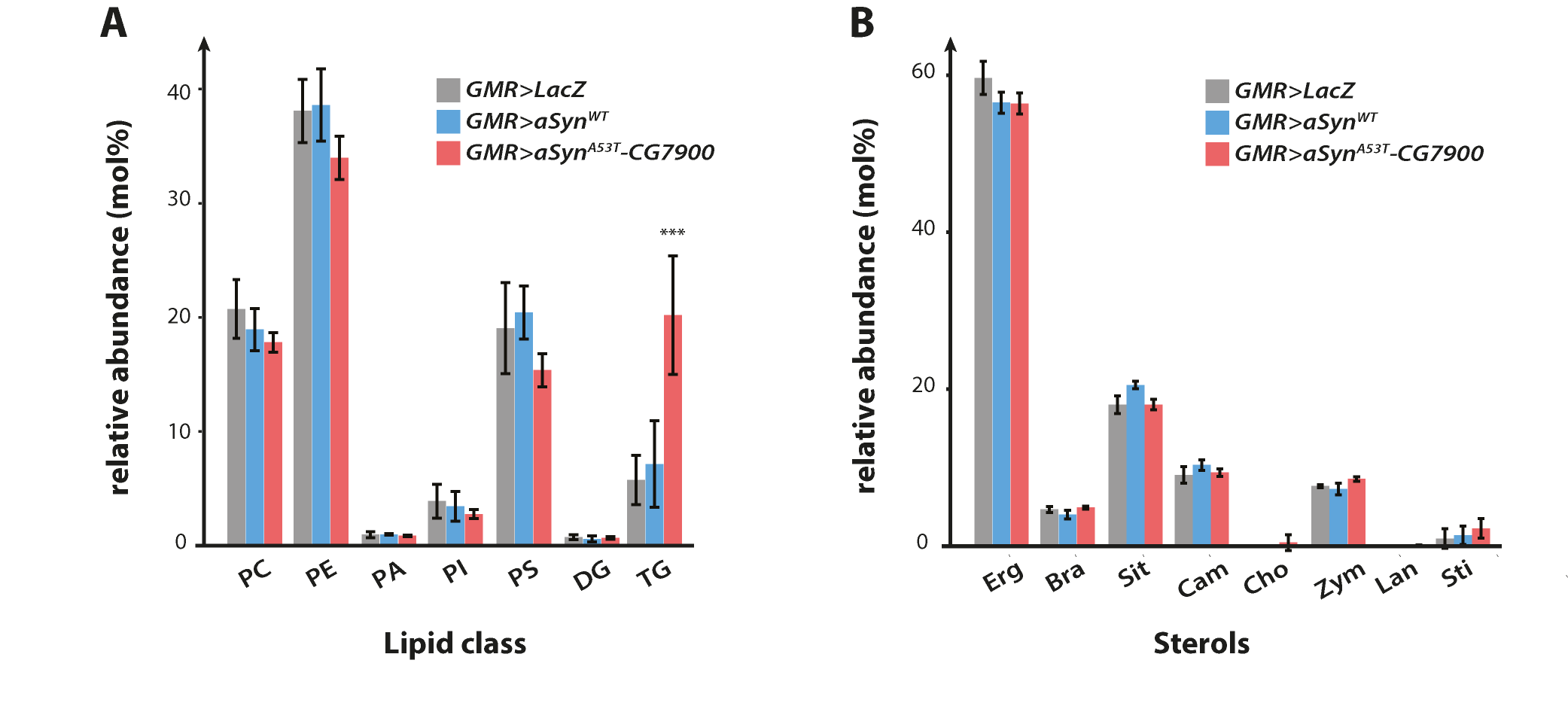

Supplement: S9 Fig — (A) Main lipid classes detected by shotgun mass spectrometric analysis of retinas from 20-day-old flies with pan-retinal expression of LacZ (control, gray bars), αSynWT (blue bars), or αSynA53T-CG7900 (red bars). Lipid quantities are expressed as mole % within each species. Data show the mean ± SD of five biological replicates. Expression of αSynA53T-CG7900 induces a significant accumulation of triacylglycerols (TG) in Drosophila retina. PC, phosphatidylcholine; PE, phosphatidylethanolamine; PA, phosphatidic acid; PI, phosphatidylinositol; PS, phosphatidyl serine; DG, diacylglycerol. (B) Sterol composition of retinas from 20-day-old_flies with pan-retinal expression of LacZ (control, gray bars), αSynWT (blue bars), or αSynA53T-CG7900 (red bars). Lipid quantities are expressed as mole % within each species. Data show the mean ± SD of 5 biological replicates. Erg, ergosterol; Bra, brassicasterol; Sit, sitosterol; Cam, campesterol; Cho, cholesterol; Zym, zymosterol; Lan, lanosterol and Sti, stigmasterol (TIF) [file pgen.1009921.s009.tif]

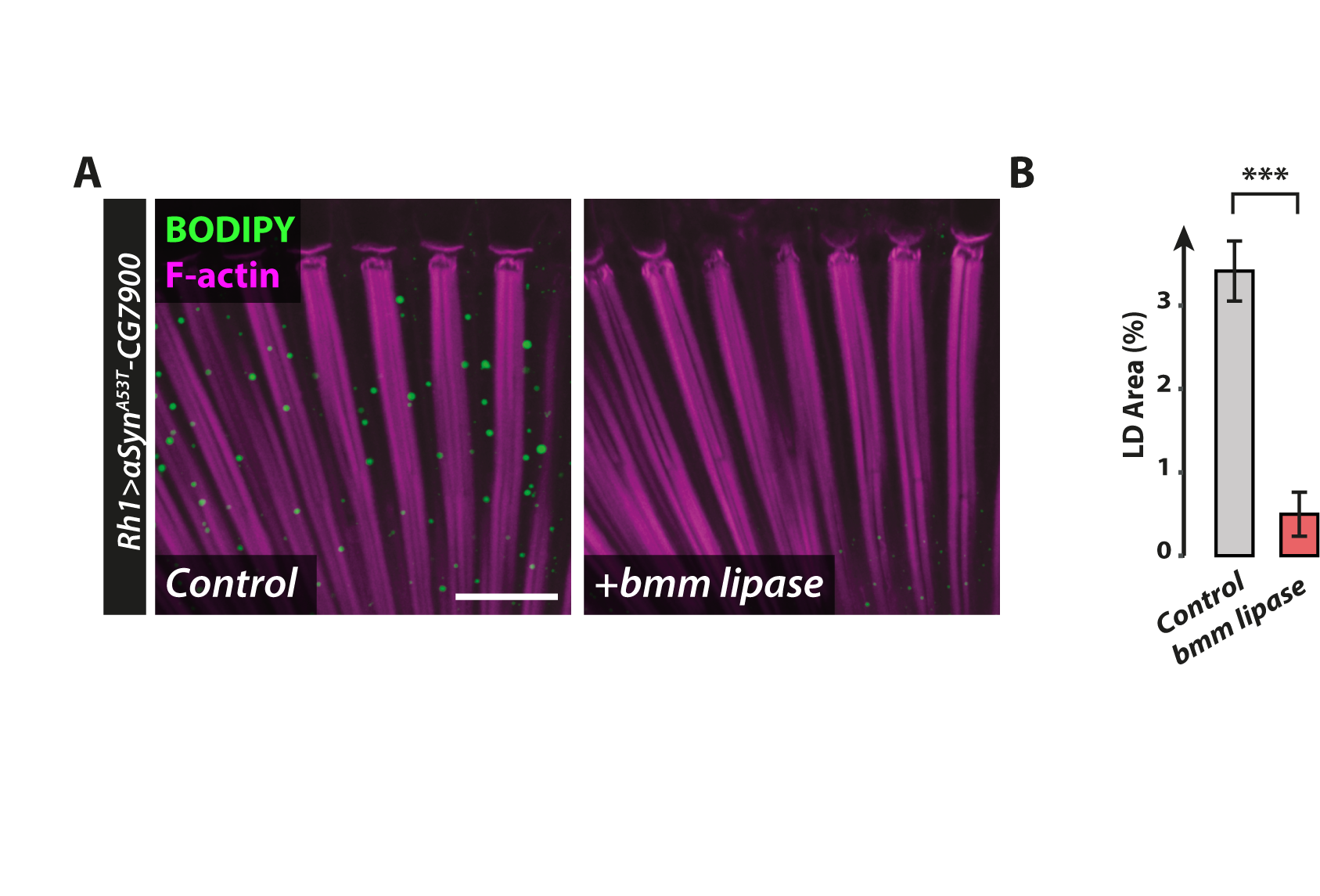

Supplement: S10 Fig — Bmm promotes the degradation of αSynA53T-CG7900 induced-LDs in Drosophila photoreceptors (A) LD staining of whole-mount retinas from flies expressing αSynA53T-CG7900 alone or in conjunction with bmm lipase in photoreceptors (Rh1-GAL4). LDs are shown in green (BODIPY) and photoreceptor rhabdomeres are in magenta (phalloidin-rhodamine). Scale bar, 20 μm. (B) Quantification of LD area from the images shown in (E). Mean ± SD. ***p<0.001 by t-test. (TIF) [file pgen.1009921.s010.tif]

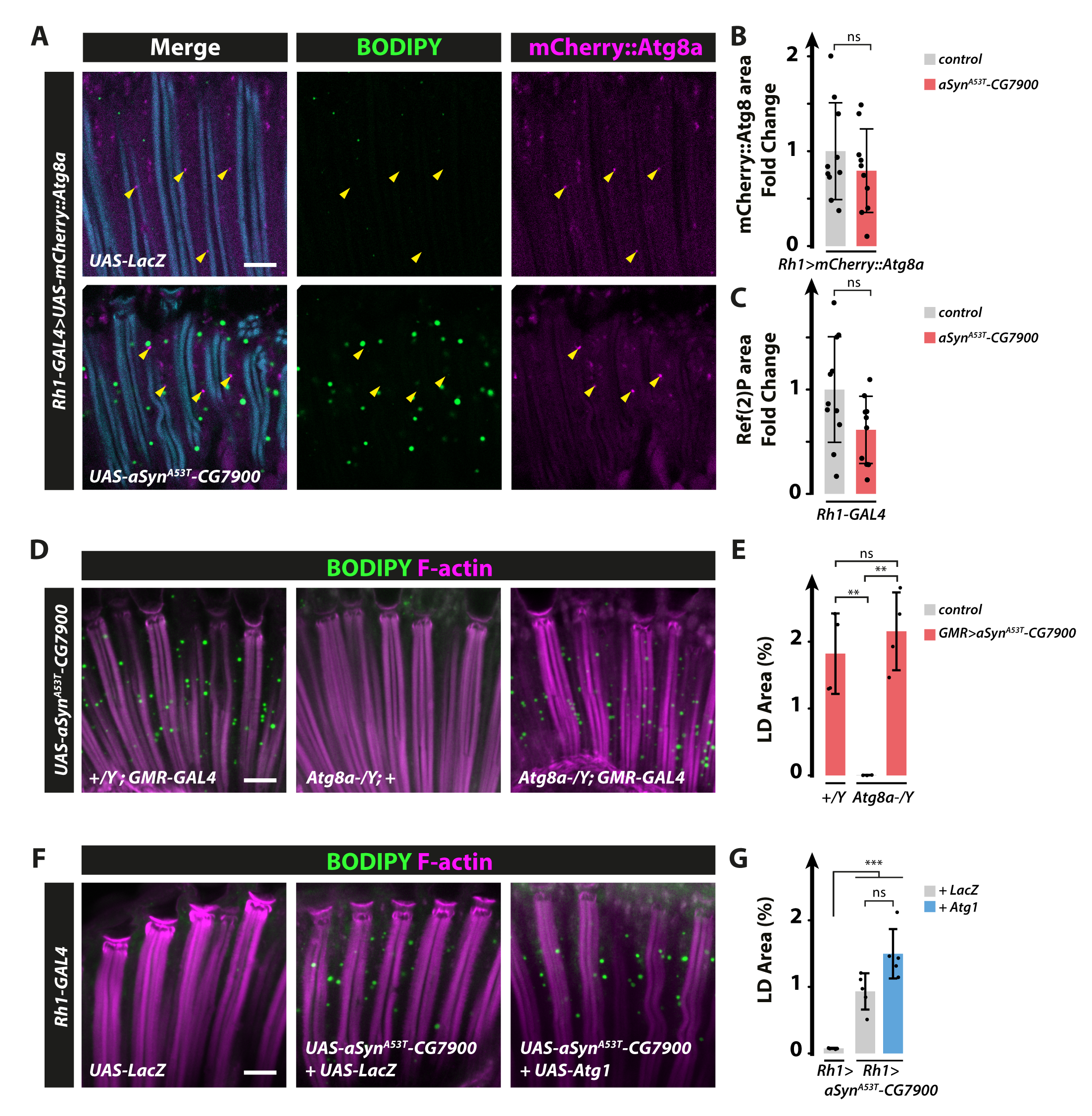

Supplement: S11 Fig — (A) LD staining of whole-mount retinas from flies expressing mCherry::Atg8a in conjunction with LacZ (Control) or αSynA53T-CG7900 in photoreceptors (Rh1-GAL4). LDs are shown in green (BODIPY), autophagosomes are in magenta (mCherry::Atg8a) and photoreceptor rhabdomeres are in cyan (phalloidin-rhodamine). Scale bar, 10 μm. (B) Quantification of mCherry::Atg8a area expressed in fold change compared to control from the images shown in (A). Mean ± SD. Ns, Non-significant by t-test. (C) Quantification of Ref(2)P punctae (anti-Ref(2)P labelling) of flies expressing LacZ (Control) or αSynA53T-CG7900 in photoreceptors (Rh1-GAL4). Expressed as fold change compared to LacZ controls. Mean ± SD. Non-significant by t-test. (D) LD staining of whole-mount retinas from Atg8aKG07569 flies expressing αSynA53T-CG7900 under the control of pan-retinal driver (GMR-GAL4). LDs are shown in green (BODIPY), and photoreceptor rhabdomeres are in cyan (phalloidin-rhodamine). Scale bar, 10 μm. (E) Quantification of LD area from the images shown in (D). Mean ± SD. ns, not significant by ANOVA. (F) LD staining of whole-mount retinas from flies expressing αSynA53T-CG7900 alone or in conjunction with Atg1 in photoreceptors (Rh1-GAL4). LDs are shown in green (BODIPY), and photoreceptor rhabdomeres are in cyan (phalloidin-rhodamine). Scale bar, 20 μm. (G) Quantification of LD area from the images shown in (F). Mean ± SD. ns, not significant by ANOVA. (TIF) [file pgen.1009921.s011.tif]
