# Supplementary material for: Abnormal accumulation of lipid droplets in neurons induces the conversion of alpha-Synuclein to proteolytic resistant forms in a Drosophila model of Parkinson’s disease
Source: PLoS Genet. 2021 Nov 17;17(11):e1009921. doi: 10.1371/journal.pgen.1009921 (PMC8635402; doi:10.1371/journal.pgen.1009921)
Supplement: Attachment — Submitted filename: PGENETICS-D-20-01524-point-by-point response.docx [file pgen.1009921.s012.docx]

**Point-by-point response to the reviewers' comments (PGENETICS-D20_01524):**

***Reviewer #1****: There is emerging evidence that lipid droplets (LDs) are deregulated in various human diseases, including Parkinson’s Disease (PD). LDs are most notably regulated by lipid metabolizing enzymes and LD-binding proteins. Here, the authors use the Drosophila eye and cultured human cells to reveal a cooperative relationship between alpha-synucleins and LD-binding proteins in promoting LD accumulation. Specifically, they show that co-overexpression of alpha-synucleins (wild type and A53T mutants) and LDs (specifically dPlins or CG7900) increase LDs. These two types of proteins co-localize on LDs. LD increases under these conditions require the lipase Bmm, and knockdown of Bmm reduces proteinase K-resistant (aggregated forms) alpha-synucleins in old flies. Based on these results, the authors propose that alpha-synucleins and LDs engage in a feed-forward loop that promotes alpha-synuclein aggregation.*

*This study’s major significance is in the revelation of a cooperative relationship between alpha-synuclein and LD-binding proteins. Overall, the main conclusions are novel and interesting. At the same time, certain aspects of the study’s design require improvements. For example, the study employs many different LD-binding proteins assuming that they exhibit the same cooperative relationship with alpha-synuclein. This assumption is not rigorously tested beyond simple LD-binding protein overexpression experiments. Control experiments are not well organized, and missing in some cases. In addition, the authors do not test if any of the observed results affect neurodegeneration. Below are specific comments along these lines for the authors to address:*

We would like to thanks the reviewer #1 for acknowledging the novelty of our work, the interesting conclusions and for his/her constructive comments, which helped improving the manuscript. In response to his/her comments, we provide **novel Figures and Figure panels** for his/her appreciation and that strengthen our conclusions, with the main points summarized here:

1) We show that the cooperative relationship between aSyn and LD binding proteins, for the accumulation of LD is not restricted to dPlin2 but now also observed with the other LD binding proteins CG7900 and LD^BD^-GFP (**Figure 5C and 5D)** and dPln1 **(Figure S5**).

2) We confirm that the accumulation of LD induced by the co-overexpression of dPlin2-GFP and aSyn takes place in photoreceptors only (**Figure 3C**).

3) We provide the requested control for the PLA assay further confirming the close proximity of aSyn and PLIN3 in SH-SY5Y cells (**Figure S6**).

4) We confirm the observation that LD accumulation in glia but not photoreceptor is dependent on Bmm with a *Bmm* mutant line (**Figure 2G**).

**Major Comments:**

***Reviewer #1 comment #1:***

*In Figure 1A, B, the authors show that dPlin1 or 2 overexpression could induce LD accumulation specifically in photoreceptor neurons. In Figure 1D, E, they show that LDs likewise increase when dPlin2 is expressed together with alpha Synuclein. Are these LDs in dPlin2 + alpha Synuclein co-expressing eyes also restricted to photoreceptor cells? The authors should add double labeling images with Na+/ K+ ATPase for Figure 1D, E, so as to make the dataset appear complete.*

In response to the reviewer’s comment, we have performed a triple labelling with Na+/K+ ATPase, dPlin2-GFP and F-actin showing clearly that LDs accumulate in the cell body of photoreceptors but not in glia cells, when dPlin2 or dPlin2 plus aSyn are overexpressed with the Rh1 driver (**Figure 3C**). This result strengthens the conclusion of a cooperative action of dPlin2 and aSyn for the accumulation of LDs in photoreceptors.

***Reviewer #1 comment #2:***

*Different Figures in the manuscript present different combinations of alpha Synucleins and LD binding proteins, with the assumption that distinct LD binding proteins may interact with alpha Synuclein in the exact same way. For example, wild type alpha Synuclein is co-expressed with dPlin2 (Figure 1), while mutant alpha Synuclein A53T is co-expressed with a different LD binding protein CG7900 (Figure 4). To allow a better comparison, the authors should add experiments where wild type and mutant alpha Synucleins are co-expressed with the same LD-binding protein, and show that these equivalent combinations lead to similar outcomes in LD accumulation.*

We agree with the reviewer that it is important to confirm that several LD binding proteins, can in cooperation with aSyn, promote LD accumulation. We now show that aSyn^WT^ enhances the accumulation of LD induced by the expression of CG7900, Klarsicht LD^BD^ (**Figure 5C, D**) and dPlin1 proteins as well (**Figure S5**). This indicates that not only dPlin2 but also distinct LD binding proteins synergistically promote the accumulation of LDs with aSyn.

Finally, the characterization of the interactions of aSyn mutated forms with LD proteins could allow further characterizations of the effect of aSyn mutations on the synergy with LD proteins on the production of LDs. However, we feel it is out of the scope of the current study.

***Reviewer #1 comment #3:***

*In Figure 1, the authors show results with dPlin2 (co-expressed with alpha-synuclein). In Figure 3, the authors now use dPlin1 overexpression as they main assay. The authors seem to assume that distinct LD-binding proteins have identical properties, but at the same time, different functions of dPlins are hinted by the authors’ comments (in page 8 the authors write that that dPlin1 generates large LDs). The authors may want to present their data more consistently by using one specific LD-binding protein (e.g. dPlin 1) in both Figures 1 and 3.*

As pointed by the reviewer, dPlin1 promotes larger LDs than dPlin2, this is why we favored dPlin1 for the analysis of the mechanisms involved in LD formation of Figure 2 in the manuscript. We also validated that the results were similar with dPlin2 (Figure S2). Moreoever, we now show that in addition to dPlin2, three additional LD binding proteins, CG7900, LD^BD^ (Figure 5C and 5D) and dPlin1 (Figure S5) have synergistic effect with aSyn on LD accumulation. Thus, all LD binding proteins tested- four in total- promote the accumulation of LDs cooperatively with aSyn. The writing of the result section has been modified to describe these novel resuts (see page 9).

***Reviewer #1 comment #4:***

*The proximity ligation assay (PLA) result shown in Figure 2C needs a negative control. The authors should include negative control PLA panel(s) where they express only one of the two interacting proteins.*

As requested by the reviewer, we now show that PLA reaction is negative if only one of the antibodies against aSyn or against PLIN3 is used in aSyn transfected SH-SY5Y cells (**Figure S6C**). The quantification of PLA foci that is now provided also shows the important increase of PLA dots that colocalize with LD stained with BODIPY. These new control experiments together with the quantification analysis demonstrate that the high PLA levels seen with SH-SY5Y cells transfected with aSyn is indeed due to the colocalization of aSyn with PLIN3.

Non-transfected SH-SY5Y cells, also exhibited some PLA foci at the LDs, but with about a ten-time reduction compared to SH-SY5Y cells transfected with aSyn (Compare quantifications **in S6B and S6D**). The dots detected with PLA in non-transfected SH-SY5Y cells are due to endogenous aSyn present at low level in these cells. This is in aggrement with a previous study showing that endogenous aSyn is immunodetected in SH-SY5Y cells (Lee et al. 2011 PMID 21886844). Thus the PLA assays, provides an interesting indirect amplification of aSyn labelling allowing to demonstrate that endogenous aSyn localizes with PLIN3 at the surface of LD in non-transfected SH-SY5Y cells. These results are now described in the result section of our manuscript (See page 8).

***Reviewer #1 comment #5:***

*The authors report negative results in dFatp or Mdy knockdown (Figure 3C, D) and draw the conclusion that “LD accumulation in photoreceptor neurons probably occurs through a mechanism independent of Bmm inhibition.” This is too strong a conclusion, as the authors cannot rule out the possibility that dFatp or Mdy knockdown efficiency was low in photoreceptors.*

In response to the reviewer’s comment, we would like first to stress that the very same RNAi against dFatp or Mdy were efficient to knock down their respective target to inhibit LD accumulation in retinal glial cells (**Supplemental Figure 3** of the initial manuscript and now **Figure A** for the reviewer). Nevertheless, we agree that is always better to support RNAi results with the corresponding mutant analyses. We now provide *dFatp* clonal and *Mdy* mutant analyses showing that LD accumulation induced by the expression of dPlin1, or dPlin2, with the photoreceptor driver Rh1 is not inhibited in *dFatp* or *Mdy* mutant cells compared to wild type tissue (**Figure 2C, 2D and 2E and Figure S2**). Thus, in contrast to glial cells, LD accumulation in photoreceptors is independent of dFatp or Mdy, indicating that a non-canonical TG synthesis mechanism is required for LD formation in photoreceptors. This statement which is considerably strengthen by our new results is now part of the revised title of the article and reflected in the different subsections of the article.

**Figure A : LDs in glia require canonical enzymes involved in TG synthesis (Fatp, Mdy).**

(A) LD staining of whole-mount retinas from flies with pan-retinal expression of *UAS-Bmm-RNAi* in conjunction with *GFP-RNAi* (control), *dFatp-RNAi*, or *Mdy-RNAi.* LDs are shown in green (Bodipy) and photoreceptor rhabdomeres are in magenta (phalloidin-rhodamine labeling of F-actin). Scale bar, 20 µm.

(B) Quantification of LD area from images shown in (A). Mean +/- SD. ***p<0.001 by ANOVA with Tukey’s HSD test.

***Reviewer #1 comment #6:***

*This study focuses on LD accumulation as a major readout of alpha Syn expression (with or without LD-binding proteins). At the same time, alpha Synuclein draws interest due to its pathological role in neurodegeneration. Do the LD-binding proteins of interest (dPlins and CG7900) affect alpha-synuclein-induced neurodegeneration? The authors speculate in the Discussion that alpha Synuclein and PLIN engage in a feed-forward mechanism that culminates in alpha Synuclein aggregation, but they don’t examine the effect on neurodegeneration. I think this is a key question that should be addressed in this manuscript.*

This is indeed an interesting question. The role of LD in neurodegeneration was not examined in our article due to the fact that the overexpression of aSyn does not induce neurodegeneration in the retina using our aSyn lines. This may be due to the fact that flies are short lived animals or that the UAS/GAL4 system used for these UAS-aSyn transgenic insertion allows aSyn expression to more physiological levels compared to other expression systems (Feany 2000, PMID: 10746727; Auluck et al. 2002, PMID: 11823645; Ordonez et al. 2018, PMID: 29249285). Instead, we focused on the pathological conversion of aSyn, detected by the proteinase K assay for which we show that the presence of LDs enhances the resistance of aSyn to proteinase K digestion. The revised version of the original manuscript also contains substantial new evidence that support our initial initial claims on the mechanisms regulating LD formation in photoreceptor neurons but unfortunately, we did not find yet the appropriate settings to test the role of LD in neurodegeneration in a fly model of Parkinson. To better reflect our results, we propose to change the title of the manuscript to: “A non-canonical lipid droplet metabolism regulates the conversion of alpha-Synuclein to proteolytic resistant forms in neurons of a *Drosophila* model of Parkinson disease”

***Reviewer #1 comment #7:***

*The authors rely on single RNAi lines for most experiments, leaving open the possibility of off-target effects. Key experiments (e.g. Bmm-RNAi or CG7900 RNAi) need to be validated through independent means.*

We now provide the analysis of *Bmm* mutant retina in which LDs were detected either by BODIPY staining or by dPlin2 staining, which validates the *Bmm RNAi* results in which the accumulation of LDs is induced in glial cells but not in photoreceptors (**Figure 2G and Figure S3**). From these results, we propose that LD accumulation in photoreceptors occurs via a Bmm-independent mechanism or that a redundant lipase is required with Bmm for LD lipolysis.

Regarding CG7900 RNAi, we showed that the accumulation of LDs induced by the combined expression of CG7900 and aSynA53T is suppressed by the expression of CG7900 RNAi (**Figure S5 of initial manuscript moved to Figure S8E and S8F of the current manuscript**). This experiment clearly shows that CG7900 RNAi suppresses the photoreceptor phenotype induced by the overexpression of CG7900, which supports that the CG7900 RNAi is efficient in photoreceptors.

***Minor comments:***

***Reviewer #1 minor comment #1:***

*In page 9, the authors write “…indicating that expression of an LD-binding protein was essential to observe the increase in LD accumulation in response to alpha Syn A53T…”*

*This sentence is somewhat incomprehensible. The authors might want to change it to “….expression of an LD-binding protein caused an increase in LD….”*

We thank the reviewer for his/her comment; this part of the manuscript has been modified as followed: “Expression of RNAi targeting *CG7900* in the *αSyn^A53T^-CG7900* transgenic line abolished the accumulation of LDs (Figure S8E and S8F), indicating that in these conditions as well, CG7900 is required for LD accumulation in photoreceptors”.

***Reviewer #1 minor comment #2:***

*In the Discussion, the authors write that “PLIN expression enhances LD content independently of Bmm lipase activity is consistent with previous data showing that neurons from ATGL-mutant mice do not accumulate LDs.” These two observations don’t appear to be consistent with each other as the mouse experiment indicates ATGL (Bmm homolog) dependency.*

Thank you. We meant that both in ATGL mutant mice, and *Bmm*-mutant *Drosophila*, neurons do not accumulate LDs which indicate that LD lipolysis may be dependent on another lipase. The paragraph regarding the existence of another lipase was clarified in the discussion (see third paragraph of the discussion on page 13).

***Reviewer #1 minor comment #3:***

*CG7900 (a homolog of FA amide hydrolase) should be given a proper name.*

This is a good suggestion, thank you. we propose to name this gene *Drosophila* fatty acid amidase 2 (dFaah2). It is now included in the manuscript on page 9.

***Reviewer #2:*** *The manuscript by Girard and colleagues describes observations that suggest a connection between lipid droplets and alpha-synuclein-mediated pathogenicity using a Drosophila system. The authors are able to confirm previous observations in other models (such as yeast and cultured mammalian cells) and provide evidence that lipid droplets somehow alter the proteinase K resistance of alpha synuclein. The proposed model of how lipid droplets and alpha synuclein synergize to promote disease is intriguing, but is by far too speculative given the sparsity of data. Overall, the manuscript is rather descriptive and a bit disjointed. There is very little mechanistic insight into the phenomena they observe, and parts of the analysis is incomplete and somewhat superficial, ignoring fundamental aspects of lipid droplet biology. In conclusion, I find the manuscript far too preliminary for publication.*

In response to the reviewer’s comments, we provide new experiments that responds to his/her main concerns regarding the gain of function, RNAi approaches and the originality of the paper. In particular, we validate our results with RNAi by using loss of function analyses with *Mdy*, *dFatp* and *Bmm* mutants (**Figures 2 and S2**). We provide a novel characterization of the LD protein CG7900 and show that similarly to dPlin2, both CG7900 and lipid-binding domain (LD^BD^) cooperate with aSyn to promote LD formation (**Figure 5**). These results answer to both “lack of novelty and disjointed focus” comments of the reviewer. Moreover, we have now detected endogenous human aSyn at the surface of LDs by PLA which emphasizes the relevance of our study on the role of aSyn at the LDs (**Figure 4C**).

***Reviewer #2 comment #1:***

*My major concerns revolve around the reliance on gain-of-function approaches, flaws in considering the mechanisms of lipid droplet formation/turnover, disjointed focus between sections, and limited originality.*

*Gain-of-function*

*There is a fundamental conceptual problem with this manuscript. Most experiments involve gain-of-function analyses, e.g. overexpression of PLIN2 and aSynWT synergize to lead to lipid droplet accumulation in photoreceptors. Although such studies can tell us what processes these proteins might affect, they do not address what this means physiologically. If overaccumulation of PLIN2 is not usually part of the pathological process, what does it tell us about aSyn pathology if PLIN2 overexpression enhances it? This is especially true since it is not clear how much relatively to wild-type PLIN1 and PLIN2 are overexpressed. Since lipid droplets have central role in lipid metabolism, it is conceivable that the physiology of these photoreceptors is quite disturbed. Some loss-of-function studies would be needed to reveal how meaningful the connection of aSyn to LDs is for aSyn pathology. The closest the authors get to this question is in Fig. 5 where they eliminate/reduce the number of lipid droplets (via Brummer overexpression) and probe aSyn protease resistance. However, without more mechanistic context the meaning of this intriguing observation is unclear.*

In most cases, we would agree with the “gain of function comment” to the exception of studies exploring the function of human pathological proteins, here aSyn, in model organisms such as *Drosophila*, yeast, or mice. Indeed, in the field of neurodegenerative diseases, the study of aSyn always requires its overexpression or the overexpression of familial mutated forms in the nervous system of the model organism. A number of important studies have used this approach and identified relevant mechanisms for Parkinson’s disease (Fanning et al. 2019, PMID 30527540; Ordonnez 2018, PMID: 29249285).

This being said, we answered to the reviewers’ by studying the endogenous aSyn and perilipin at the LD and provide a new evidence that endogenous human aSyn can be detected by proximity ligation assay at the surface of LD in human neuroblastoma SH-SY5Y cells (**Figure 4C**). This result is novel and strengthens our conclusion about the importance of the binding of aSyn to LDs in human neurons (see the end of second paragraph of the discussion on page 13).

We agree with the reviewer that it is interesting to determine if the accumulation of dPlin2 or another LD binding protein could contribute to the pathological process induced by aSyn. In support of this hypothesis, it has been shown that perilipins accumulate in aging tissues (Yan et al. 2017, PMID: 28966044, Shimabukuro et al. 2016, PMID: 27029648). This could lead to an appropriate cellular context for LD accumulation in elderly individuals which in turn may affect the progression of Parkinson’s disease. We feel this analysis opens the ground for future work but it is out of the scope of the current study. In current submission, we have modified the discussion to better reflect this perspective of our work (see penultimate paragraphe of the discussion on page 14).

***Reviewer #2 comment #2:***

*The authors also need to discuss the apparent discrepancy to the literature (Fanning et al., 2019), where lipid droplets protect against aSyn toxicity while their data suggest the opposite.*

In response to the reviewer’s comment, we have clarified the existing paragraph of the discussion about the work of the Selkoe lab (see page page 14).

“Our results show that LDs contribute to αSyn conversion to proteinase K resistant forms, which indicates that LDs may be involved in the progression of PD pathology. This is an apparent discrepancy with the results in Fanning et al. (2019), in which LDs protect from lipotoxicity cells expressing αSyn. In this study the authors used cellular models including yeast cells, and rat cortical neuron primary cultures exposed or not to oleic acid. In such cellular context, they propose that αSyn induces the accumulation of toxic diacylglycerol (DG), which is subsequently converted to TG and sequestered into LDs. LDs are thus protective by allowing the sequestration of toxic lipids. In our study, realized in the fly retina, αSyn expression did not induce TG accumulation. In *Drosophila* nervous system, toxic DG may not reach sufficient level to promote photoreceptor toxicity. Interestingly, this difference allowed us to study the binding of αSyn to LD and examine their contribution to pathological conversion of αSyn, which was not explored in Fanning *et al.*. Indeed, our results suggest an alternative but not mutually exclusive role for LDs in promoting αSyn misfolding and conversion to a proteinase K-resistant forms. The increased LD surface could provide a physical platform for αSyn deposition and conversion. In support of this hypothesis, it was previously proposed that αSyn aggregation is facilitated in the presence of synthetic phospholipid vesicles (Galvagnion et al., 2015, PMID: 25643172).”

***Reviewer #2 comment #3:***

*Mechanism of lipid droplet biogenesis and turnover*

*In Fig. 3, the authors try to unravel the mechanism by which PLIN1 overexpression in photoreceptor cells results in lipid droplet accumulation. Surprisingly, knockdown of brummer in photoreceptors does not counteract accumulation, suggesting that the accumulation is not due to inhibition of this lipase; based on the literature, this had been a reasonable leading hypothesis. However, the authors do not investigate whether their knockdown of brummer was actually successful in photoreceptors. Their controls only establish that brummer knockdown worked in glial cells. If their driver is not strong enough or if the dsRNA used is for some reason not as effective in photoreceptors, then the experiment is not interpretable. It would be necessary to independently confirm efficacy of knockdown.*

The following statement of the reviewer is apparently not correct: “*Surprisingly, knockdown of brummer in photoreceptors does not counteract accumulation”*, probably the reviewer meant: “knockdown of brummer in photoreceptor is not sufficient to induce LD accumulation”, which is the opposite.

To answer to the reviewer’s comment and support the results obtained with dsRNA against Bmm, we now show that LDs accumulate in glial cells but not in photoreceptors in *Bmm* mutant retina (**Figure 2G and Figure S3**). Collectively, these results allow us to demonstrate that *Bmm*-mediated lipolysis is not regulating LD content in photoreceptors.

***Reviewer #2 comment #4:***

*But even if the result were true, it is unclear what that means mechanistically. As the authors themselves acknowledge in the discussion, a different lipase might be acting; indeed, there is another proposed triglyceride lipase (dob, doppelgänger of brummer) in flies, and in mammals, there is a neuron-specific triglyceride lipase (Inloes et al., 2014). The authors also do not consider the well-established alternative lysosomal turnover of lipid droplets, lipophagy.*

We are glad that the reviewer agrees with our discussion on the point that another lipase might be acting in photoreceptors. As suggested, we assessed the role of dob (Doppelganger of brummer), which despite its name, is not reported as a TAG lipase. We found that knock-down of dob did not lead to LD accumulation in photoreceptors, suggesting that dob is not the missing photoreceptor lipase (data not shown). We would be happy to show this result upon request.

In response to the next reviewer’s comment, we agree that autophagy in the form of chaperone-mediated autophagy (CMA) or lipophagy may be considered. Interestingly, it was shown that CMA-mediated degradation of PLIN2 and PLIN3 is an alternative putative mechanism regulating LD level (Kaushik and Cuervo, 2015). In our hands, mutations in the autophagy gene, Atg8, did not lead to LD accumulation in *Drosophila* retina (data not shown), thus a more systematic analysis will be required to identify the proteolytic mechanisms regulating PLINs degradation and LD accumulation in neurons. We have now discussed a possible role of CMA-mediated degradation of perilipin, that may contribute to the degradation of LD in neurons (see penultimate paragraph of the discussion page 14).

***Reviewer #2 comment #5:***

*In search of mechanism, the authors also knock down the enzyme DGAT1 (Mdy) which catalyzes triglyceride synthesis. Again, they do not verify that Mdy knockdown is effective in photoreceptors, making is impossible to come to solid conclusions when they do not see a lipid droplet phenotype. In addition, there is a distinct enzyme, DGAT2, that produces triglycerides. Without also investigating DGAT2, the conclusion that de novo TG synthesis is not involved is not valid.*

To answer the reviewers’ comment, we now provide novel experiments showing that LD accumulation induced by the expression of dPlin1 is not affected in *dFatp* mutant clones or DGAT1 (Mdy) mutant retina (**Figure 2C, 2D and 2E**). Regarding DGAT2, we could not assess its role because DGAT2 is triplicated in the fly genome and so far there is no triple mutant available. We propose to modify our statement as followed: These results indicate that PLIN-induced LD accumulation in photoreceptor neurons occurs through a mechanism independent of *dFatp*- and *Mdy*-mediated *de novo* TG synthesis and is thus distinct from the mechanism of LD accumulation in glial cells (see first sub-section of the Results section).

***Reviewer #2 comment #6:***

*The authors also do not consider another alternative: lipid droplets do not only store triglycerides, but also sterol esters. Might the lipid droplets accumulating upon PLIN overexpression be mainly made up of sterol esters? If so, an entirely different set of enzymes for synthesis and turnover would be involved. Curiously, the lipidomic analysis in Fig. S6 does not list sterols and sterol esters even though they are mentioned in the materials and methods.*

This is a good point from the reviewer, thank you, indeed LDs can be made of TAG or sterol esters. We now provide a lipidomic analysis showing that TAG but not cholesterol esters are up regulated upon expression of aSynA53T plus CG7900 (**Figure S9**). This result comforts the model that TAG-containing LDs recruit aSyn and promote its pathological conversion.

***Reviewer #2 comment #7:***

*Finally, the authors propose “stabilizing LD structure” as the mechanism to explain why lipid droplets accumulate. The authors seem to see this as a distinct mechanism, but I do not have any idea how that would work, and they do not offer an explanation. It is certainly quite interesting that overexpression of two unrelated lipid droplet proteins also causes lipid droplet accumulation. I suspect that all those proteins act by suppressing lipid droplet turnover, coating the lipid droplet surface and thereby preventing access of a particular lipase that in photoreceptors usually eliminates lipid droplets.*

*The stabilization hypothesis is not supported by data, it is easier to think of an unknow lipase no?*

We would like to thank the reviewer for this comment.

The possibility that lipolysis is controlled by an unknow lipase was proposed as a possibility in our discussion but we initially considered “stabilization” induced by the expression of LD binding protein as a strong hypothesis. The point raised by the reviewer that the stabilization hypothesis is technically difficult to demonstrate is valid, especially *in vivo*. In response to the reviewer’s comment, we propose to only keep the stabilization hypothesis in the discussion but to remove it from the summary and from the schematic model (**Figure 6** of the initial manuscript). Instead, we propose to include in the model the hypothesis of an unknown lipase (**Figure 7** of the new submission and the last paragraph of the discussion).

***Reviewer #2 comment #8:***

*Disjointed approach*

*In Figs. 1 and 2, the authors show that dPLIN2 overexpression synergizes with aSyn, and that the two proteins both localize to lipid droplets. However, in Fig. 3C, they look at lipid droplet biosynthesis using dPLIN1 which produces a much stronger phenotype than dPLIN2. It is not clear whether anything they learn from the dPLIN1 analysis will be relevant for the aSyn analysis as they have not shown a genetic interaction or colocalization between PLIN1 and aSyn. They simply seem to assume that PLIN1 and PLIN2 act by the same mechanism, which is not warranted. Similarly, they could use LD[BD] and CG7900 overexpression to test for genetic interactions with aSyn. The results of such studies might reveal if aSyn somehow interacts with anything that causes lipid droplet accumulation or if PLIN2 plays a specific role.*

This point was raised by reviewer 1 as well. In response to the reviewers’ comment, we now confirm that not only dPlin1 but also dPlin2 induces LD accumulation independently of dFatp or Mdy (**Figure 2C, 2D and Figure S2**). Furthermore other LD binding proteins can, in cooperation with aSyn, promote LD accumulation. We now show that aSynWT enhances the accumulation of LD-induced by the expression of dPlin1, CG7900 or the Klarsicht lipid droplet binding domain (LD^BD^) as well (**Figure 5C, 5D and Figure S5**). These results indicate that not only dPlin2 but also distinct LD binding proteins cooperatively promote the accumulation of LDs. Thus, so far, all LD binding proteins tested promote LD accumulation cooperatively with aSyn. Collectively, our results support that the overexpression of LD proteins leads to the accumulation of LD in photoreceptors which involve non canonical mechanism independent of dFatp and Mdy enzymes.

***Reviewer #2 comment #9:***

*In Fig. 4 and 5, the authors take advantage of the fortuitous insertion of their aSyn[A53T] transgene into the promoter of CG7900, allowing co-overexpression. However, this set up complicates the interpretation of their experiments. They show, using RNAi, that CG7900 is necessary for lipid droplet accumulation in the co-overexpressing flies. But there is no quantitative comparison (e.g. does the mutant aSyn protein synergize with CG7900 to cause even more lipid droplets to accumulate?), and the effect on protease resistance in Fig. 5 might in part be due to a combinatorial effect with CG7900. As there is little insight given into what CG7900 does mechanistically, it makes the meaning of their results difficult to ascertain: we have to compare the wild type aSyn to the mutant plus CG7900, so differences cannot be confidently attributed to either the mutant or CG7900. For example, in Fig. S6, the increase in TG is observed in the co-overexpressing line. Maybe this is simply a phenotype of CG7900 overexpression and has nothing to do with aSyn.*

The point of whether CG7900 can synergize with aSyn to promote LD accumulation was also asked above and we now provide the requested result (**Figure 5C and 5D**). Based on this result, we predict that TAG accumulation induced by CG7900 will be enhanced upon aSyn addition.

The next point is about proteinase K resistance, which was increased in the line expressing both aSyn^A53T^ and CG7900. We agree that it would be great to show also the result combining independent transgenic lines expressing, such as aSyn^A53T^ and CG7900. However, due to time constraints, we have not done this experiments. We hope that the additional evidence provided in Figure 6 will be sufficient to convince the reviewer.

***Reviewer #2 comment #10:***

*Originality*

*The originality of the findings is also limited. Fig. 1 establishes their experimental system; although to my knowledge it has not previously been shown that PLIN1 or PLIN2 overexpression causes lipid droplet accumulation in photoreceptors, such an effect is not entirely surprising as that’s what is observed in other cell types. The synergy with aSyn is interesting, but maybe also not terribly surprising since aSyn has been linked to increased LD accumulation in yeast and mammalian cells. The colocalization data in Fig. 2 are good, but recruitment of aSyn to lipid droplets was previously observed in mammalian cells (Cole et al. 2002) and in vitro (Thiam et al., 2013), so again it is not altogether surprising. Fig. 3 has nothing to do with aSyn, but attempts to understand the mechanism of lipid droplet accumulation upon dPLIN1 overexpression. As described above, this analysis is incomplete and flawed.*

The revised version of the manuscript, which includes further confirmations of our initial data allows us to claim that PLIN-induced lipid droplet formation occurs via a non-canonical mechanism in photoreceptor neurons. This is a novel and original finding that is now reflected in all the sections of the manuscript including the title.

In addition, we would like to argue that study of LD accumulation in neurons and their role in an *in vivo* model of Parkinson’s disease is very original.

In the last few years, it was realized that contrary to common belief, LDs are not mere fat deposits and besides their primary energetic function, *i.e.* to provide fatty acids for beta oxidation at the mitochondria (mostly in fat tissues where LDs were originally studied). Indeed LDs have additional functions in stress response, including in the nervous system as it was shown in several recent key studies, including ours (Bailey et al. 2015 PMID: 26451484, Liu et al. 2015 PMID: 25594180, Liu et al. 2017 PMID: 28965825, Van den Brink et al. 2018 PMID: 30199545, and reviewed in Welte and Gould, 2017 PMID: 28735096). However, those studies have focused on the accumulation of LDs in glial cells, and none have investigated the production and function of LDs in neurons despite the fact that LDs are observed in neurologic pathological conditions. Here, we provide the first study investigating the process of LD formation in neurons and its consequences on pathological progression in aSyn *Drosophila* model of Parkinson’s disease. In addition, if LD accumulation was observed in several cellular models expressing aSyn including yeast, rodent and human cells (Cole et al. 2002 PMID: 11744721, Outeiro and Lindquist 2003 PMID: 14657500, Fanning et al. 2019 PMID 30527540) so far LDs were not observed yet in any model organism expressing aSyn. Thus it makes the fly model a unique system to explore the process of LDs *in vivo* in neurons and its consequence in a well-recognized model of Parkinson’s disease. A more detailed introduction is now provided to better describe the literature and the novelty of our work (See penultimate paragraph of the introduction).

***Reviewer #2 comment #11:***

*Fig. 4 is original, but provides limited insight since all the phenotypes described depend on the gene CG7900 which is not further characterized.*

We are glad that the reviewer acknowledges the originality of Figure 4. In response to his/her comments, we now provide an additional characterization of CG7900 localization at the LD in both *Drosophila* photoreceptors and fat body (**Figure 5A and 5B**) and show that CG7900 and aSyn cooperatively promote LD accumulation in photoreceptors (**Figure 5C and 5D**). Those data which are presented as revised subsection of the result section (page 9), demonstrate that CG7900 is a conserved *bonafide* LD protein in *Drosophila*, which LD-binding properties are conserved in human cells. The study of CG7900 FAAH enzymatic activity would be interesting to study but we think it is out of the scope of this study. Indeed, the fact that the cooperative role with aSyn of all LD binding proteins tested in our study (dPlin1, dPlin2, CG7900 and LD binding domain of Klarsicht) strongly suggest that the cooperative role of LD proteins with aSyn is due to a steric effect of the LD binding protein at the LD.

***Reviewer #2 comment #12:***

*That leaves Fig. 5 as the main original contribution, but the mechanism underlying these observations is not clear. For example, the presence/absence of lipid droplets likely results in profound physiological changes in the cell (e.g. forcing lipid droplet turnover with brummer overexpression may increase ROS via elevated beta oxidation or may lead to heightened fatty acid signaling); it might be these indirect downstream effects that alter aSyn protease resistance, not the lipid droplet surface.*

The question of whether LDs have a direct role in aSyn resistance to proteinase K is a valid point, that needs to be thoroughly discussed. First, the fact that decreased LDs by expression of the lipase Bmm or conversely the accumulation of LDs induced by expression of dPlin2 and aSyn support that aSyn proteinase K resistance is dependent on LD level. This last result is now strengthens by our new experiment showing that dPlin2 induces a clear enhancement of aSyn resistance to proteinase K. (**Figure 6A and 6B**). This points to a direct mechanism involving aSyn at the LD surface. This is also supported by the observation that aSyn is found at the LD surface when LD formation is primed by LD binding proteins in both *Drosophila* and SH-SY5Y cells. Nevertheless, to answer the reviewer’s comment, we have added the following sentence in the discussion (page 12): “Although our results point to a direct role of LDs on αSyn resistance to proteinase K, we cannot exclude an indirect mechanism by which autophagy or reactive oxygen species levels would contribute to this phenomenon.”

***Reviewer #2 comment #13:***

*Finally, in the model in Fig. 6., the increased levels of inclusion bodies/aggregates are nicely hypothesized, but there is no direct observation of those structures in the paper. So, the model remains quite speculative.*

We agree with the reviewer that we did not directly detect aSyn aggregates, which are usually difficult to detect *in vivo*. Our **Figure 7** model is based not only on our result but also on previous observations showing that αSyn aggregation is facilitated in the presence of synthetic phospholipid vesicles (Galvagnion et al., 2015, PMID: 25643172) as it is now described in our discussion (page 14)

**In addition, I have the following minor concerns:**

***Reviewer #2 minor comment #1:***

*In the figures where %LD area is calculated, I could not find a description how many images the data were based on and how many different animals those images represent. This should either be clearly indicated either in the methods or in each figure.*

For all figures, a minimum of 6 retina per experiments, repeated three times representing at least 18 different flies were analyzed. This information will be added in the material and method section of the manuscript.

***Reviewer #2 minor comment #2:***

*On page 3, the authors use the term “peridroplet proteins” to describe what is ordinarily called lipid droplet proteins. This is a non-standard term that an internet search revealed to be only use in one other publication, once. It certainly is not used in the review the authors cite as support for their statement. The authors are certainly free to make up their own nomenclature, but – if so – they should clearly define this new term.*

We thank the reviewer for this comment, we have changed peridroplet to lipid droplet proteins (LD proteins) which provides a clearer definition.

***Reviewer #2 minor comment #3:***

*At the bottom of page 3, the authors claim that both human PLIN1 and dPLIN1 are restricted to adipose tissues. This is not correct for either protein, and the review cited does not claim this. For example, dPLIN1 has been shown by Men et al., 2016, to be expressed in the wing disc.*

We thank the reviewer for his/her comment and we have modified the manuscript (see top of page 3 in the Introduction) accordingly with “dPlin1 is *mostly* restricted to adipose tissues but was also found expressed in Drosophila wing disc (Men et al. 2016).”

***Reviewer #2 minor comment #4:***

*Fig. 2C: It seems the PLA experiment needs a negative control, such as cells not transfected with aSyn. It is also not clear how to interpret this experiment. Would any two lipid droplet proteins interact in this assay? If so, this experiment does not provide any additional information beyond Fig. 2B. Or do the authors want to conclude that aSyn and PLIN3 have a special, more intimate association? If so, they have to show that unrelated lipid droplet proteins do not give signal in this assay.*

In response to the reviewer’s comment, we now show that PLA reaction is negative if only one of the antibodies against aSyn or against PLIN3 is used in aSyn transfected SH-SY5Y cells (**Figure S6C**). The quantification that is also provided shows the important increase of PLA dots that often colocalize with LD stained with BODIPY. These new control experiments together with the quantification analysis demonstrate that the high PLA levels seen with SH-SY5Y cells transfected with aSyn is indeed due to the colocalization of aSyn with PLIN3.

Non-transfected SH-SY5Y cells, also exhibited some PLA foci at the LDs, but with about a ten-time reduction compared to SH-SY5Y cells transfected with aSyn (Compare quantifications **in S6B and S6D**). The dots detected with PLA in non-transfected SH-SY5Y cells are due to endogenous aSyn present at low level in this condition. Thus the PLA assays, provides an interesting indirect amplification of aSyn labelling allowing to demonstrate that endogenous aSyn localizes with PLIN3 at the surface of LD in non-transfected SH-SY5Y cells. The result section (page 6) and discussion (page 13) have been changed to reflect this finding.

***Reviewer #2 minor comment #5****:*

*Fig. 5B: How often were these experiments repeated? Statistics for whether the observed differences are significant should be provided. Fig. S7B lacks error bars and statistics*

In response to the reviewer’s comment, we have quantified the difference in PK resistance of Fig5B (now in **Figure 6D**). We have also repeated the experiments assessing PK resistance in flies overexpressing dPlin2 and aSyn (presented in S7B of the original manuscript), which confirm our initial observation, showing unambigously that the expression of dPlin increases the resistance of aSyn to PK digestion. This results, which include the statistics, has been moved to the main figure (**Figure 6A and 6B**). Collectively, through two independent means, our results provide evidence that LD increase aSyn resistance to proteolytic digestion by PK.

***Reviewer #3****: Girard et al., investigated lipid metabolism in neurons with a particular emphasis on the interplay between the alpha-Synuclein protein and lipid droplets in the light of Parkinsons Disease (PD). As model systems, they used the photoreceptor neurons of Drosophila and a neuroblastoma cell line. They demonstrate that the overexpression of LD proteins such as the Perilipins induce LDs in the neurons and that alpha-Synuclein targets (some of) these LDs. Finally, the authors demonstrate that LDs might protect alpha-Synuclein from proteolytic cleavage. The experiments are mostly convincing while some controls (as outlined below) are missing or the specificity of tools – the GMR-Gal4 activator line – are probably less clear as presented (also see below). In essence, the question I kept asking myself was whether the authors could indeed show that “neuronal lipid droplets promote a pathological conversion in alpha-synuclein via a feed-forward mechanism” which is the*

*title of the manuscript. At this stage, I unfortunately think that the study is too preliminary to make this statement. Together with the other shortcomings outlined below, I find the manuscript is therefore not strong enough to allow publication in PLOS Genetics.*

We would like to thank the reviewer for her/his comments, which helped us improving the manuscript. We are glad that he/she found our experiments mostly convincing and we are now providing additional experiments supporting our claims in the point by point-response to his/her comments. Here summarized our main answers to his/her comments:

- we would like to thank the reviewer for his/her suggestion to study the localization of endogenous aSyn using PLA, which is difficult due to low levels in SH-SY5Y cells. Using this approach, we now report that endogenous human aSyn is found at the surface of LDs in SH-SY5Y cells (**Figure 4 and S6**). This is an important result, which provides a strong rationale to our study on the role of LD in aSyn pathology.
- As requested by the reviewer, we provide evidence that LD accumulation in glial cell is cell autonomously induced by knock down of *Bmm* using the glial 54C driver and in Bmm mutant retina (**Figure 2**).
- A point raised by the reviewer is that the title of the manuscript does not best reflect the data presented. The choice of this title was explained in the text associated with the model figure (**Figure 7 of the manuscript**). Nevertheless, we agree that a different title could better reflect the results presented in our study, we propose the following novel title: “**A non-canonical lipid droplet metabolism regulates the conversion of alpha-Synuclein to proteolytic resistant forms in neurons of a *Drosophila* model of Parkinson disease”**
- We finally apologize to the reviewer if she/he felt that previous work was not sufficiently quoted. We now provide the requested citations in the text of the manuscript.

**Major comments:**

***Reviewer #3 comment #1:***

*The potential to induce lipid droplets by the overexpression of Perilipins and other LD-associated proteins is well described. This overwhelming body of data is not acknowledged sufficiently.*

We agree that the role of perilipins and other LD-associated proteins in the formation of LDs is well described. We have now updated the introduction with a more detailed description on LD biogenesis and the role of perilipins and quoted several additional references (see Introduciton page 3).

***Reviewer #3 comment #2:***

*The reasons for the LD accumulation are not totally clear. The authors tested for an impact on lipolysis or lipogenesis (please see my comments below). My guess is that in their experimental system, lipid droplets are too small to be detected under basal conditions but become stabilized and enlarged by the overexpression of the LD-associated proteins. Perhaps also the ER is affected and the authors might want to investigate this.*

First, we agree with the reviewer that small existing LDs could become enlarged by the expression of LD proteins. This hypothesis is supported by our EM observations showing that photoreceptors have few small LDs (Van den Brink et al. 2018 PMID 3019954). Second, regarding a possible tight association of ER and LDs due to a defective budding that could explain the enlargment of LDs, we have looked again to our electronic microscopy ultrastructural analyses in which LDs were induced by expression of aSyn^A53T^ and CG7900. Large LDs were found in the cytoplasm but not associated with the ER, which suggests that the budding of LD from the ER occurs normally (data not shown and available upon request).

***Reviewer #3 comment #3:***

*More importantly, however, the information we have concerning the link between alpha-Synuclein and LDs almost exclusively relies on overexpression studies (the authors might want to mention some more details concerning the binding of alpha-Synuclein to LDs demonstrated e.g. in* [*https://doi.org/10.1016/j.devcel.2019.05.003*](https://doi.org/10.1016/j.devcel.2019.05.003) *or*

[*https://doi.org/10.1073/pnas.1307685110*](https://doi.org/10.1073/pnas.1307685110) *or summarized in* [*https://doi.org/10.3389/fncel.2019.00175*](https://doi.org/10.3389/fncel.2019.00175)*). An important piece of evidence for the relevance of their findings would be to show the localization of endogenous alpha-Synuclein on LDs (see below) or at least the demonstration of an overexpression of Perilipin or similar LD proteins in Parkinson’s disease patients, which could support their hypothesis.*

We agree that we could have quoted more extensively the studies reporting the binding of aSyn to LD and studies reporting that aSyn expression promotes TAG and LD accumulation (Cole et al. 2002 PMID: 11744721, Outeiro and Lindquist 2003 PMID: 14657500, Chorlay and Thiam 2020, PMID: [32328636](https://www.ncbi.nlm.nih.gov/pubmed/32328636); Thiam et al. 2013, PMID: [23901109](https://www.ncbi.nlm.nih.gov/pubmed/23901109); Alza et al. 2019, PMID: 31118888, Sanchez-Campos et al., 2018 PMID: 30098984) (See novel introduction, page 4 and 5). As pointed by the reviewer, all these studies rely on gain of aSyn function in mammalian cell culture or in yeast cells and the presence of endogenous aSyn at the LD remains to be studied. To answer to this point, we used the PLA assay as suggested by the reviewer, which presumably detects and amplifies the interaction of single molecule. PLA assays using anti-aSyn and anti-PLIN3 antibodies on non-transfected SH-SY5Y cells revealed a consistent PLA staining which colocalized with BODIPY staining (**Figure 4C-D**). This result is the first to examine and show that endogenous aSyn binds LD. This supports that the binding of aSyn to LD is relevant in PD. We agree that identification of the upregulation of LD proteins in PD patients would be very valuable as well. We will think to include these analyses in our future studies but it is out of the scope of the current study.

***Reviewer #3 comment #4:***

*Further, the authors invoke with their title the impression, that lipid droplets provide an explanation for the pathological conversion of alpha-Synuclein. Yet, I do not find there is sufficient evidence for this conclusion in the presented manuscript. Is the protein forming more aggregates in the presence of LDs? Is the structure changed? All what is shown is a relatively weak evidence for an effect on the protease accessibility.*

The reviewer has a point, additional experiments would be required to demonstrate that aSyn resistance to proteinase K is also associated with additional hallmarks, such as aggregates formation or change of aSyn structure. Unfortunately, the detection of aSyn aggregates is usually difficult in *Drosophila* (Pocas et al. 2014 PMID 25452431) and were not observed in our model. This may be due to the fact that *Drosophila* are short lived animals, which is fine to detect the first step of aSyn conversion revealed by the proteinase K assay but not appropriate to look at aggregates accumulation. In response to his/her comment we have modified the title of our article to better reflect our results: **A non-canonical lipid droplet metabolism regulates the conversion of alpha-Synuclein to proteolytic resistant forms in neurons of a *Drosophila* model of Parkinson disease.**

***Reviewer #3 comment #5:***

*In Figure 2, the distribution of the overexpressed alpha-Synuclein is very different in the neuroblastoma cell line and the photoreceptors. While in the fly, the protein is almost everywhere and only some of the dPLIN2 decorated LDs also show an enrichment of alpha-Synuclein, in the neuroblastoma cell line alpha-Synuclein is almost exclusive localized to (all of) the LDs with almost no background. Is the endogenous alpha-Synuclein protein also found at LDs? I understand that the protein amount might be too low for an immunofluorescence detection (even the PLA was performed with transfected cells, correct?).*

We thank the reviewer for his/her comment, the detection of endogenous aSyn at the LD was possible with the PLA assay (**Figure 4D**). You may see response to the reviewers’ comment above (comment 3).

***Reviewer #3 comment #6:***

*Yet, a purification of the LDs from the neuroblastoma cells with sucrose gradients and subsequent western blot should be able to detect endogenous protein, which would help to support the importance of the findings.*

This a good idea which could help detecting endogenous LD in the LD fraction. We have not realized this experiment but we hope that the PLA assay detecting endogenous aSyn and PLIN3 at the LD will convince the reviewer (**Figure 4D**).

***Reviewer #3 comment #7***

*The LD localization of alpha-Synuclein was shown before multiple times. Thus, the rationale to do the co-localization experiments (Fig. 2A) should be introduced differently.*

We agree that the rational to realize PLA assay could be presented differently (moved from Figure 2A to Figure 4A). Now that the PLA assay shows that endogenous aSyn binds LD we have used this to better introduce this experiment (See result section on page 8).

***Reviewer #3 comment #8:***

*The brummer lipolysis line of arguments are not clear to me. The authors demonstrate that under basal conditions there are no LDs in the photoreceptor neurons. The experiments with the GMR-Gal4 driven bmm RNAi surprisingly do show an LD accumulation while also in the glial cells under basal conditions no LDs were detected.*

*These results potentially imply that in the glial cells a futile cycle of LD biogenesis and degradation could exist. As an alternative explanation, however, the GMR-Gal4 driver could also be just less specific than the authors present. Indeed, several publications (e.g. PMID:22911584 or PMID:26440079) demonstrate an GMR-Gal4 induced expression in many more tissues. Thus, the effects observed by the authors could also have many other possible causes (e.g. a misregulated lipid metabolism in another tissue which results in ectopic lipid deposits in the glia). Based on the difficulties to interpret the results due to the lack of controls (e.g. different Gal4 driver line / direct test for an impact on lipolysis / monitoring of the expression of the driver in aged flies,…), Figures 3A and 3B should be removed from the manuscript. Otherwise, the authors need to present more convincing data including the appropriate controls.*

We would like to thanks the reviewer for this suggestion. The hypothesis of systemic effect of *Bmm RNA*i expressed with GMR is a possibility. To verify the requirement of Bmm in glial cells we have used the glial cell driver 54C (**Figure 2F**) that we previously used in a former study (Van den Brink et al. 2018 PMID: 30199545). We also assessed LD accumulation in Bmm mutant flies (**Figure 2G and S3**). Using the 54C driver to knock down *Bmm*, or the *Bmm* mutant retina, we found the same glial pattern of LDs accumulation than with GMR, which further supports our claim that the Bmm lipase is required in glial cells but not in photoreceptors for LD turnover (**Figure 2F and 2G and S3**).

***Reviewer #3 comment #9:***

*The conclusion: “It also suggests that αSyn and dPlin1/2 could not induce LD degradation through a mechanism involving lipolysis.” is also not clear to me.*

We apologize if this sentence was not clear. The paragraph describing these results has been changed to better reflect our novel results on the mechanisms by which perilipin induces the accumulation of LDs in neurons (first paragraph on page 7).

*The data shown in Fig. S3 is similarly affected by the potentially broad(er) expression of the GMR-Gal4 driver line. Did the authors check the overall TAG content of the flies? A broad impact might even translate to altered organismic TAG levels.*

We did not test systemic TAG levels with the GMR-GAL4 driver, instead we used the glial specific driver 54C and Bmm mutant retina above (**Figure 2F and 2G and S3**). We hope the reviewer will be convinced with these new results.

***Reviewer #3 comment #10:***

*The lipogenesis targeted experiments are also not without problems. To start, one can argue whether dFATP is really a canonical enzyme in TAG synthesis and the way the authors word it, it seems that dFATP and DGAT1/Midway are the major (only) enzymes. dFATP is a fatty acid transport protein, which apparently also has acyl-CoA synthetase activity (PMID: 16477381). The authors should explain this more. Currently, only the function of DGAT1 is spelled out.*

In response to the reviewer’ comment, we included a more detailed description on dFatp, which is a fatty acid transport protein but also contains acyl CoA activity involved in anabolism and catabolism of lipids (Dourlen et al. 2015, PMID: 26416577) (See second paragraph of the introduction). Work in C. elegans, *Drosophila* and mice have shown that it plays a central role for TAG synthesis and LD formation (Xu et al., PMID: 22927462; Van den Brink et al. 2018 PMID: 30199545). The fact that the knockdown of *Mdy* and *dFatp* (or *dFatp* mutant clones and *Mdy* mutant) inhibits LD in glia but not in photoreceptors indicate that neuronal LD use a distinct mechanism independent of these canonical enzymes (Figure 2C, 2D and Figure S2).

***Reviewer #3 comment #11:***

*Overall, the results are puzzling, as the lipids need to come from somewhere. In order to truly rule out a stabilization of emerging LDs / lipid synthesis, the authors would need to test also other LD biogenesis / lipogenesis proteins (e.g. DGAT2, fatty acid synthase, seipin, GPAT4 just to name a few).*

Fifteen candidate genes involved in lipid synthesis or transport (including seipin, GPAT4, FASN…) were tested by dsRNA for a role in LD accumulation induced by aSyn^A53T^ plus CG7900, but none could modify the accumulation of LDs (data available upon request). We envisage a unbiased genetic screen to find regulators of LD accumulation in photoreceptors but this is out of the scope of the current study.

***Reviewer #3 comment #12:***

*What happens if the authors incubate the photoreceptor cells with exogenous lipids such as fluorescently labeled C12 fatty acids (available e.g. from Thermo / Molecular Probes)? Are lipids from the outside incorporated?*

This is an interesting experiment that would allow to show that exogenous lipid can cross photoreceptor membranes to label LDs. Unfortunately, due to time constraints and the important number of priority experiments needed we have not done these experiments.

***Reviewer #3 comment #13:***

*The results of the authors using the LD binding domain or CG7900 overexpression indeed show that overexpressed LD proteins can induce the stabilization of LDs. Yet, the LDs need to come from somewhere and the experiments presented to rule out an importance of lipolysis or lipogenesis are not convincing and lack controls. Thus, this section should get toned down significantly.*

We agree with the reviewer, the possibility that lipolysis is controlled by an unknow lipase was proposed in our discussion but we considered “stabilization” induced by the expression of LD binding proteins as a strong hypothesis. In response to the reviewer’s comment, we propose to keep the stabilization hypothesis only in the discussion but to remove it from the summary and from the model (**Figure 7**). Instead, we propose to include the an unknown lipase as the main hypothesis (see last paragraph of the introduction).

***Reviewer #3 comment #14:***

*Fig. 4 does not add much knowledge other than introducing the A53T mutation of alpha-Synuclein. I suggest to incorporate the findings with the comparable previous figures. The headline of the section “Co-expression of alpha-Synuclein A53T and CG7900 proteins induces LD accumulation in Drosophila photoreceptor neurons” is also misleading, as the authors demonstrate that the LD accumulation is only based on the CG7900 overexpression (“Expression of RNAi targeting CG7900 in the aSynA53T-CG7900 transgenic line abolished the accumulation of LDs (Figure S5).”). Thus, the headline is inappropriate. Please change accordingly.*

*Perform separately CG7900 and aSynA53T*

We agree with the reviewer that the data on CG7900 and aSyn^A53T^ could be introduced differently. We now provide a substantial additional characterization of CG7900 which demonstrates that it is a bonafide LD protein (**Figure 5A and 5B**). Moreover, we showed that similarly to dPlin2, CG7900 cooperates with aSyn for LD formation (**Figure 5C and 5D**). These novel data will allow us to introduce Figure 4 by focusing on CG7900, a novel LD protein cooperating with aSyn for LD induction.

***Reviewer #3 comment #15:***

*The proteinase K stability experiments are intriguing and might point in a direction that LDs could stabilize and/or protect alpha-Synuclein. The data presented, however, are not totally convincing to me. In Figure S7, the protein loading in the Synuclein / dPLIN2 co-expression is much lower and I am having a hard time to come to the same conclusions as the authors e.g. looking at the quantifications shown in panel B. If I only look at the highest Proteinase K amount, the amount of Synuclein protein left is really difficult to compare based on the varying loadings. Do the authors have blots with more similar loading amounts? Why is in Fig. 5 the 2 mg Proteinase K amount quantified whereas in Fig. S7 the 1.5 mg Proteinase K condition is quantified? Please show the quantification for all Proteinase K amounts. Why is the age-dependent difference not seen in Fig. S7? Is this specific to the mutant form? The authors should provide more information.*

In response to the reviewer’s comments, we have repeated the proteinase K (PK) assay on flies expressing aSyn^WT^ and dPlin2. We came to the same conclusion that dPlin2 addition increases the resistance of aSyn to proteinase K digestion with more clear differences which are shown with the quantification (**Figure 6 A and 6B**).

In some occasions the difference in PK sensitivity was more clearly visible, when comparing 1.5µg or 2µg of proteinase K. This may be due to some variability in the resistance of aSyn to PK digestion between aSynWT and aSynA53T, which was solved by performing the dose response in all these experiments. Despite these little differences, we were able to faithfully reproduce the presented phenotype in several independent experiments (see supplemental file 1).

***Reviewer #3 comment #16:***

*The rationale for / conclusion of the experiment shown in Fig 1C is unclear. The Gal4 line used by the authors was specific for the neurons. Thus, why should lipid accumulation or protein expression happen in the glia? Please explain / rephrase in the text. Also show the expression domain of the driver line using e.g. the driver together with a UAS controlled cytosolic GFP transgene (and the antibody stainings for glia specificity).*

This is due to the fact that LDs are commonly observed and studied in glial cells but rarely in neurons in physiological or pathological conditions (Van Den Brink et al., 2018; Liu et al. 2015; Bayley et al. 2015). We have clarified this point in the first paragraph of the result section. We have also showed the specificity of the Rh1 driver and the localization of LD in photoreceptor neurons with ATPase staining (**Figure 1C and D**).

**Minor points:**

***Reviewer #3 minor comment #1:***

*In their description of LD biogenesis in the introduction important factors are missing and the situation is oversimplified. At least Seipin as a key factor should be mentioned.*

We have updated the introduction with a more detailed paragraph on LD biogenesis, including the role of Seipin (see second paragraph of the Introduction).

***Reviewer #3 minor comment #2:***

*The two PLIN proteins in flies do not only have redundant functions – the situation is actually more complicated (doi: 10.1242/jcs.101329). Please rephrase.*

Thank you for pointing this out. We have rephrased this sentence as followed on page 3 of the Introduction: … “the *Drosophila* genome encodes two PLINs, Lsd-1 and Lsd-2 (hereafter named dPlin1 and dPlin2) that play distinct roles in LD homeostasis (Heier and Kühnlein, 2018)*.* dPlin2 is indeed considered as the guardian of LDs by shielding LD away from lipases, while dPlin1 by interacting directly with lipases can either stimulate or inhibit lipolysis (Beller et al., 2006, 2010; Bi et al., 2012; Grönke et al., 2003).”

***Reviewer #3 minor comment #3:***

*In the introduction, the link between alpha-Synuclein as well as PD and lipids needs to be further explained. As is, this is too short and the lay user will not be able to understand the rationale of the performed experiments (in the abstract, the authors mention that “Parkinson’s disease is…characterized…by abnormalities in lipid storage”). Please summarize what is known concerning altered lipid metabolism in PD patients and how (we think) alpha-Synuclein is linked to lipid droplets and affects lipid metabolism.*

We would like to thank the reviewer for this comment and we have included a more detailed review of the literature on the link between aSyn, Parkinson and lipids and quoted the appropriate references (Introduction on page 4).

***Reviewer #3 minor comment #4:***

*The term “LD membrane” might be misleading as LDs are not shielded by the classical phospholipid bilayer (“membrane” usually refers to this structure) but a phospholipid monolayer instead. Please use a different term.*

Thank you we will change to LD phospholipid monolayer.

***Reviewer #3 minor comment #5:***

*Is the sole increase in TAG shown in Fig. S6 not surprising? Intuitively I would guess that such a prominent increase in TAG also requires an increase in phospholipids for the packaging of the neutral lipids. Please comment.*

This is an interesting comment thank you. During the LD accumulation process, one could indeed expect an increase of phospholipids concomitant with TAG. The fact that a phospholipid increase was not detected in the lipidomic analysis (Figure S9) could suggest that phospholipids come from reticulum membrane, which are already synthetized. Alternatively, additional phospholipid synthesis may not be necessary if the binding of LD proteins to LD can fulfill the function of phospholipid, stabilizing the LDs as it has been proposed for PLIN4 (Čopič 2018 PMID: 29626194).

--------------------

<b>Have all data underlying the figures and results presented in the manuscript been provided?</b> <br />

Large-scale datasets should be made available via a public repository as described in the <i>PLOS Genetics</i> <a href="<http://journals.plos.org/plosgenetics/s/data-availability>" target="_blank">data availability policy</a>, and numerical data that underlies graphs or summary statistics should be provided in spreadsheet form as supporting information.

Reviewer #1: Yes

Reviewer #2: Yes

Reviewer #3: No: I did not find a spreadsheet providing the numerical data that underlies the plots or summarizing statistics.

We now provide the supplemental file 1 for new version of the manuscript.

--------------------
