# Supplementary material for: Abnormal accumulation of lipid droplets in neurons induces the conversion of alpha-Synuclein to proteolytic resistant forms in a Drosophila model of Parkinson’s disease
Source: PLoS Genet. 2021 Nov 17;17(11):e1009921. doi: 10.1371/journal.pgen.1009921 (PMC8635402; doi:10.1371/journal.pgen.1009921)
Supplement: Attachment — Submitted filename: Response to reviewer Girard et al. Rev2.docx [file pgen.1009921.s013.docx]

**Point-by-point responses to the reviewers’ comments (Girard et al. )**

**Reviewer #1**: This is a revised manuscript reporting that Lipid Droplet (LD) coating proteins synergize with alpha synucleins to promote LD accumulation in neurons, and that LDs protect alpha synucleins from proteolytic digestion, a property associated with alpha synuclein aggregation in human cells. Through the revision process, the authors have satisfactorily addressed points raised during the initial review cycle. Among others, they added new data showing that different sets of LD binding proteins have similar properties in synergizing with alpha synucleins. With the newly added data, the organization of the manuscript has significantly improved. Although the authors did not experimentally address the effect of LDs on alpha synuclein-induced neurodegeneration, they now highlight LD's effect on the pathological conversion of alpha synuclein to a proteinase K resistant form. Accordingly, they changed the title reflecting this focus.

This is a fine study and I support the publication of this manuscript.

I noticed a few grammar mistakes in the revised manuscript text, and I recommend fixing those errors prior to publication.

We are glad that the reviewer #1 is satisfied with this version of the manuscript, we would like to thank him for his time and contribution.

We have fixed several grammar mistakes in the revised version of the manuscript.

**Reviewer #2**: In their resubmitted manuscript, Girard et al. targeted most, yet not all of my comments and issues raised in the initial submission. Unfortunately, I find not all of the arguments or newly presented data fully convincing. While I appreciate the efforts made, I continued to ask myself whether the authors could make the claim presented in the title of the manuscript (the title changed now to: “A non-canonical lipid droplet metabolism regulates the conversion of alpha-Synuclein to proteolytic resistant forms in neurons of a Drosophila model of Parkinson disease”). The authors actually leave it in my opinion open what the “non-canonical lipid droplet metabolism” really means. They tested a (the?) very prominent lipase and two enzymes important for neutral lipid deposition. Yet, other processes, such as autophagy and more specifically lipophagy remain untouched (there is one remark that Atg8 perturbation did not affect the phenotype). Thus, a key aspect of the study – how the LDs form and potentially affect the alpha-synuclein function – remains unanswered. As this is a central point of the paper, this needs to be addressed before publishing the article can be considered at this level.

We agree with the reviewer that lipophagy could regulate the content of LD in photoreceptors expressing aSyn and LD binding proteins. To evaluate lipophagy, we examined the co-localization of autophagic vacuoles and LDs as previously reported (Jung et al. 2017, PMID 28507162). For that, we tested if the autophagy mCherry::Atg8a reporter construct co-localizes with LD in photoreceptors expressing both aSynA53T and the LD protein CG7900 (Figure S11). We observed that BODIPY-labelled LDs, which accumulate following the co-expression of aSynA53T and CG7900, did not co-localize with the few mCherry::Atg8a punctae (Figure S11A). Furthermore, there was no apparent difference mCherry::Atg8a punctae in photoreceptor expressing aSynA53T-CG7900 compared to control condition (Figure S11B). We also examined Ref(2)P protein accumulation, which is used as proxy for the autophagy flux (Robin et al. 2019 PMID 30563404). We found not difference in Ref(2)P in photoreceptor expressing αSynA53T-CG7900 compared to wild type retina (Figure S11C). This suggests that basal autophagy is not affected by the expression of aSynA53T-CG7900 and that lipophagy is very rare or absent in these conditions. Next, we asked if the inhibition or activation of autophagy could lead to a difference in the level of LDs in photoreceptors expressing aSynA53T-CG7900. For that we examined LDs in photoreceptors of Atg8a mutants or photoreceptors over-expressing Atg1. We found no difference of the accumulation of LDs induced by the expression of aSynA53T-CG7900 under these conditions compared to controls (Figure S11D-G). Collectively, these results indicate that lipophagy is not a major factor in the regulation of LDs induced by the expression of aSynA53T and CG7900. In response to the reviewer’s comment, we have included a new supplementary figure (Figure S11), and updated the text in the result section (pages 10-11). The new data, which exclude lipophagy, provide additional circumstantial evidence that non-canonical mechanisms regulate LD turnover in neurons. However, we agree that the mechanisms regulating LD turnover in photoreceptor neurons remain elusive. Accordingly, we replaced the terms “non-canonical metabolism” by “abnormal accumulation” and "non-canonical mechanisms" by "alternative mechanisms".

Here is the proposed modified title for the manuscript:

« Abnormal accumulation of lipid droplets in neurons induces the conversion of alpha-Synuclein to proteolytic resistant forms in a *Drosophila* model of Parkinson disease »

As outlined above, a key point is the hypothesis that an unknown lipase degrades LDs in neurons in a futile cycle and that this lipase is different from Brummer. The authors base this statement on the data shown in Figures 2 and S3. In order to test whether lipogenesis is required for the neuronal LD deposition, they co-express the LD-associated protein(s) and target / use mutants of the anabolic enzymes midway and FATP2, respectively. In the corresponding lipase loss-of-function experiments, however, they omit the LD protein co-expression. What is the rationale behind this? In neurons, there might be just not enough coating protein(s) available to stabilize the lipids in droplets without the presence of additional protein (which leaves the situation somewhat artificial; the supplemental data uses antibody staining to detect endogenous Lsd-2 which is indeed lacking in the neurons). Thus, the statement that a different lipase should be required for the build-up of droplets is not clear to me and only one possible explanation. I understand that the overexpression of the LD proteins alone results in the appearance of LDs; yet in case a lipase (brummer?) is involved, its knock-down should result in even larger LDs. The authors thus generate hypotheses (including the ill-defined “non-canonical lipid droplet metabolism”), which are not really fully supported by their data.

We thank the reviewer for her/his concern, related to a possible role of Bmm in photoreceptor LD control, which cannot be uncovered by loss-of-function analysis due to lack LD protein packing capacity in these neurons. This is particular relevant in light of a recent study by Wat et al. 2020 (PMID 31961851), in which the authors suggest a role for *bmm* in the somatic cells of the gonad and in neurons to regulate systemic TG. As requested by the reviewer, we now show that *bmm* knockdown in the context of dPlin2 overexpression did not increase LD cumulative area compared to dPlin2 overexpression alone (**Figure 2G-H**). Accordingly, at least in photoreceptor neurons subject to LD protein overexpression, endogenous Bmm does not regulate LD breakdown. However, *bmm* over-expression clears LDs from photoreceptors co-overexpressing aSynA53T and CG7900 (Figure S10). This provides evidence that photoreceptor LDs are responsive to control by lipases and suggests that another unknown lipase may play such function.

We have included this novel result in the manuscript and propose the following paragraph of the discussion (page 13):

“A recent study, showed that Bmm plays a role in the somatic cells of the gonad and in neurons to regulate systemic TG breakdown (Wat et al., 2020). The authors also suggested that Bmm may also play a role in regulating LD turnover in neurons, although this was not directly tested in this study. Our results using *bmm* knock-down and *bmm* mutant do not support a role of Bmm in the regulation of LD content in photoreceptor neurons. However, we cannot exclude the possibility that Bmm would be required in subpopulation of neurons to regulate LD content but this would require further analyses.”

The authors mention that they tested a collection of lipogenic gene functions for their role in the LD deposition (information from the rebuttal letter). They should add this data to the manuscript given the appropriate controls (e.g. knock-down efficacy) are present to further strengthen their point.

In response to the reviewer’s comment, we now provide the results showing the effect of candidate genes involved in lipid synthesis or transport were tested by dsRNA for a role in LD accumulation induced by aSyn^A53T^ plus CG7900 (**Figure I**). None of the tested candidate could modify the accumulation of LDs, which suggests that alternative mechanisms are required to regulate LD content in photoreceptors. We propose to keep this additional information as a figure to the reviewer only.

We envisage an unbiased genetic screen to find regulators of LD accumulation in photoreceptors but this is out of the scope of the current study.

**Figure I: aSynA53T-CG7900 expression induces LD in photoreceptors independently the main LD biogenesis actors.**

Quantification of LD area relative to control (GMR>aSynA53T-CG7900 + GFP-RNAi). Flies expressing aSynA53T-CG7900 in conjunction with RNAi targeting genes involved in lipogenesis or lipid transport. RNAi targeting Outsider, Silnoon, Basigin, Glaz, Nlaz, CS and ACC were validated in Liu et al. 2017, PMID: 28965825). dPlin2-RNAi was validated in Bailey et al. 2015, PMID: 26451484. *mdy*-RNAi was validated in Van den Brink et al. 2018, PMID 30199545 and using loss of function (this study); LPCAT-RNAi was validated in Moessinger et al. 2014, PMID: 25491198. RNAi targeting DGAT2/CG1942, CG1941 were validated in Wicker-Thomas et al. 2015, PMID: 26353752. RNAi targeting CG1946, GPAT4, minotaur were not tested previously. Mean ± SD. Not significant, P>0.05 by ANOVA with Tukey’s HSD test.

Also, the authors unfortunately decided against following my suggestion to provide fluorescent fatty acids to test for an importance of exogenous lipid supply for the build-up of the LDs, which I still think could have helped to better test the mechanism of the LD origin. Further, they include in their model exogenous fatty acids as source for the LD deposition but not really test this. As an alternative to my initially suggested experiment, the authors could also test what happens if the amount of circulating free fatty acids is increased e.g. by an overexpression of brummer in the fat body. Given that they already use brummer overexpression (Fig. S10), this should be a feasible experiment.

In response to the reviewer’s comment, we have tested if Bmm-dependent TG mobilization form in the fat body is sufficient to promote the accumulation of LDs in photoreceptors. We over-expressed *bmm* under the control of the fat body driver Lpp-Gal4 and stained LD with BODIPY in adult retina. As shown previously (Grönke et al., 2005), flies with excessive Bmm expression in fat body are lean compared to controls (see attached **figure for the reviewer IIA**). However, TG mobilization from the fat body is insufficient to promote LD accumulation in neither photoreceptor nor glial cells of the retina (**Figure IIB**, **IIC**). This suggests that excess circulating FAs alone is not sufficient to drive LD accumulation in the retina. However, we cannot exclude that in condition in which dPlin2 would be overexpressed in photoreceptors, circulating FAs could have contributed to LD formation. This experiment could not be realized in the time-frame of this revision given that it would require a complex genetic, such as combining two binary expression systems UAS/GAL4 and QUAS/QF to achieve the dual expression of *bmm* in fat body and dplin2 in photoreceptors. Considering that the answer to the reviewers’ point could not be fully addressed, we propose to show below the results of this experiment as a figure to the rewiever only (**Figure II**). In response to the reviewer’s comment to better reflect that the origin of FAs involved for LD formation is still unknown, we have removed the arrow indicating the exogenous origin of the FAs in the figure model (Figure 7).

**Figure II: Overexpression of *bmm* in the fat body does not induce accumulation of LD in the retina.** A- Quantification of whole fly triglycerides (TG) measured using a Coupled colorimetric assay (CCA, Hildebrandt et al. 2011 PMID 21931614). *** p<0.001 by ANOVA with Tukey’s HSD test. B- LD staining of whole-mount retinas from flies expressing *bmm* in fat body (Lpp-GAL4). LDs are in green (BODIPY) and photoreceptor rhabdomeres are in magenta (phalloidin-rhodamine). Scale bar, 10 µm. C- Quantification of LD area expressed as % of total retinal area. Data are from the images shown in (B). Mean +/- SD. Ns, not significant by ANOVA with Tukey’s HSD test.

Concerning the “substantial additional characterization of CG7900 which demonstrates that it is a *bona fide* LD protein (Figure 5A and 5B)” (quoted from the rebuttal letter) I am not that convinced concerning the substantiality of characterization. The fat body is densely packed with LDs and accordingly, the admittingly circular signal might indicate LD localization. Yet, it could also be close apposition of e.g. the ER and to really show the LD localization I would expect e.g. a biochemical LD purification and western blotting to demonstrate the co-purification of the protein.

We thank the reviewer for this comment. We agree that ER-LD contact sites are a source of concern for immunolocalization and biochemical analysis alike. Yet, they are typically not covering all the LD surface, for which reason we consider closed ring-like structures decorating the LD core as strong evidence for a LD-binding protein. To further support this conclusion in the case of CG7900 we now provide additional experimental evidences. We expressed CG7900::GFP in the *Drosophila* larval fat body and co-labelled CG7900::GFP with dPlin1, which is an obligatory LD protein in fat body (Beller et al., 2010, PMID 21035762). We observed that CG7900 co-localizes with dPlin1 at the surface of LD (Figure 5B). To further assess CG7900 localization at the LD in a cellular context, where LDs are less densely packed, we heterologously expressed a CG7900::RFP in HeLa cells in which endogenous LD are were visualized with BODIPY staining. We observed that the surface of all LD are CG7900-positives (Figure 5C). Collectively these results substantiate our observation that CG7900 is a bona fide LD binding protein.

Taken together, I still think the study is lacking significant experiments to support the presented hypotheses and conclusions and thus I do not recommend its publication in PLoS Genetics at this point of time.

We thank the reviewer for her/his constructive comments and we hope she/he will be convinced that the additional experimental evidence combined with the more balanced interpretation of findings of the revised version of the manuscript will now justify the publication of this study in PLoS Genetics.

**Have all data underlying the figures and results presented in the manuscript been provided?**
Large-scale datasets should be made available via a public repository as described in the *PLOS Genetics* [data availability policy](http://journals.plos.org/plosgenetics/s/data-availability), and numerical data that underlies graphs or summary statistics should be provided in spreadsheet form as supporting information.

Reviewer #1: Yes

Reviewer #2: Yes

PLOS authors have the option to publish the peer review history of their article ([what does this mean?](https://journals.plos.org/plosgenetics/s/editorial-and-peer-review-process#loc-peer-review-history)). If published, this will include your full peer review and any attached files.


If you choose “no”, your identity will remain anonymous but your review may still be made public.


**Do you want your identity to be public for this peer review?** For information about this choice, including consent withdrawal, please see our [Privacy Policy](https://www.plos.org/privacy-policy).

Reviewer #1: **Yes:** Hyung Don Ryoo

Reviewer #2: No
